# Regulatory Genomic Circuitry of Brain Age by Integrative Functional Genomic Analyses

**DOI:** 10.1093/gpbjnl/qzaf064

**Published:** 2025-08-08

**Authors:** Xingzhong Zhao, Anyi Yang, Jing Ding, Yucheng T Yang, Xing-Ming Zhao

**Affiliations:** Huzhou Central Hospital, Affiliated Central Hospital Huzhou University, Huzhou 313000, China; Department of Neurology, Zhongshan Hospital and Institute of Science and Technology for Brain-Inspired Intelligence, Fudan University, Shanghai 200433, China; Department of Neurology, Zhongshan Hospital and Institute of Science and Technology for Brain-Inspired Intelligence, Fudan University, Shanghai 200433, China; Department of Neurology, Zhongshan Hospital and Institute of Science and Technology for Brain-Inspired Intelligence, Fudan University, Shanghai 200433, China; Huzhou Central Hospital, Affiliated Central Hospital Huzhou University, Huzhou 313000, China; Department of Neurology, Zhongshan Hospital and Institute of Science and Technology for Brain-Inspired Intelligence, Fudan University, Shanghai 200433, China; MOE Key Laboratory of Computational Neuroscience and Brain-Inspired Intelligence, and MOE Frontiers Center for Brain Science, Fudan University, Shanghai 200433, China; Huzhou Central Hospital, Affiliated Central Hospital Huzhou University, Huzhou 313000, China; Department of Neurology, Zhongshan Hospital and Institute of Science and Technology for Brain-Inspired Intelligence, Fudan University, Shanghai 200433, China; MOE Key Laboratory of Computational Neuroscience and Brain-Inspired Intelligence, and MOE Frontiers Center for Brain Science, Fudan University, Shanghai 200433, China; State Key Laboratory of Medical Neurobiology, Institutes of Brain Science, Fudan University, Shanghai 200032, China

**Keywords:** Brain aging, Brain age gap, Genome-wide association study, Gene regulatory network, Brain disorder

## Abstract

Brain age gap (BAG) is a valuable biomarker for evaluating brain healthy status and detecting age-associated cognitive degeneration. However, the genetic architecture of BAG and the underlying mechanisms are poorly understood. Here, we estimated brain age from magnetic resonance imaging with improved accuracy using our proposed adversarial convolution network (ACN), and applied the ACN model to an elderly cohort from the UK Biobank. The genetic heritability of BAG was significantly enriched in regulatory regions and implicated in glial cells. We prioritized a set of BAG-associated genes, and further characterized their expression patterns across brain cell types and regions. Two BAG-associated genes, *RUNX2* and *KLF3*, were found to be associated with epigenetic clock and diverse aging-related biological pathways. Finally, two BAG-associated hub transcription factor genes, *KLF3* and *SOX10*, were identified as regulators of pleiotropic risk genes for diverse brain disorders. Altogether, we improve the estimation of BAG, and identify BAG-associated genes and regulatory networks implicated in brain disorders.

## Graphical abstract

**Figure:**
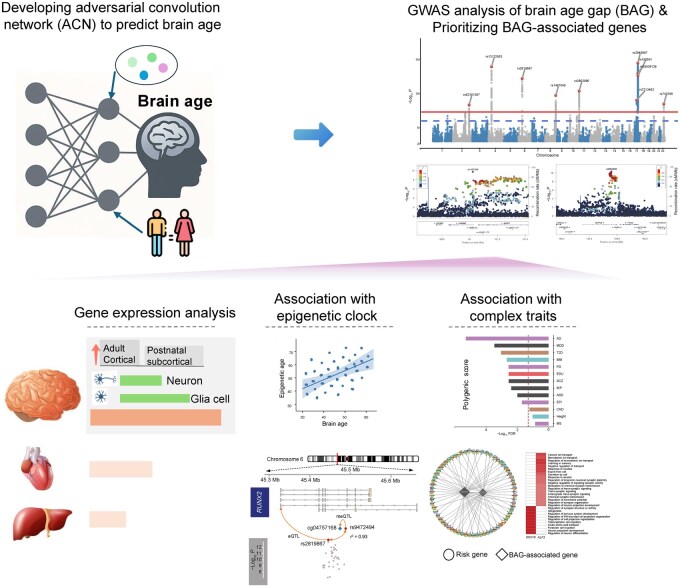

## Introduction

As a complex biological phenomenon, brain aging is characterized by the progressive accumulation of molecular and cellular damage over an individual’s lifetime and instigates the emergence of chronic brain diseases and cognitive decline [1–3]. Unveiling the molecular mechanisms underpinning brain aging is critical for preserving brain health [2]. Structural magnetic resonance imaging (MRI) has proven to be a valuable technology for quantitatively assessing the aging status of the brain [4], while machine learning and deep learning methodologies have been utilized to predict “brain age” by extracting aging-related features from structured MRI images [5–7]. The difference between brain age and chronological age, termed the brain age gap (BAG), can be used to discern whether an individual’s brain appears older or younger than that of an age-matched healthy one. BAG has been proven as a novel biomarker for evaluating the degree of brain aging [6].

Previous studies have established the associations between BAG and cognitive impairments, as well as psychiatric disorders [8–10]. For example, patients diagnosed with Alzheimer’s disease (AD) show notably larger BAG scores than healthy participants, consistent with the observation that AD can expedite the process of brain aging [11,12]. BAG has been revealed to be heritable with a heritability score of ∼ 0.2 [13,14]. BAG also shows polygenic overlap with diverse psychiatric disorders, such as schizophrenia (SCZ) and bipolar disorder (BIP) [13,14]. BAG provides a potent medium to bridge brain aging and brain disorders, making it necessary to systematically explore their shared genetic architecture and molecular mechanisms.

Brain structure undergoes intricate modifications in cellular senescence and molecular pathways during aging [15,16]. Transcriptomic data from brain cells and tissues at different developmental stages can be employed to identify genes associated with brain aging by comparing gene expression profiles across different stages [13,17,18]. For example, Buckley et al. revealed that the transcriptomic signatures from microglia can accurately estimate the degree of brain aging [19]. Kang et al. showed that the decline in synaptic functions during brain aging can be triggered by the increased expression of *REST* and decreased expression of *TP73* [18]. However, the association between the molecular signatures and the dynamics of brain structures during brain aging remains unclear. The extent to which BAG represents the brain aging process and its association with molecular changes during brain aging need to be clarified. The availability of large amounts of brain imaging data and functional genomic data from the human brain provides an excellent opportunity to explore these questions.

In this study, we characterized the genetic architecture of BAG by developing an adversarial convolution network (ACN) with improved generality and accuracy in brain age prediction, and then applying the ACN model to an elderly cohort from the UK Biobank (UKB). We found that the genetic heritability of BAG was significantly enriched in the regulatory regions and implicated in glial cells. Nine novel significant variants and 44 novel BAG-associated genes were prioritized. We showed that the BAG-associated genes exhibited elevated expression levels in aging-related brain regions and cell types. Interestingly, BAG showed concordant patterns with the epigenetic clock and aging-related biological pathways. Furthermore, we identified two BAG-associated hub transcription factor (TF) genes, *KLF3* and *SOX10*, regulating pleiotropic risk genes for brain disorders. The target genes of *KLF3* and *SOX10* were enriched in neuroinflammation-related functions. To summarize, our study improves the methodology of BAG estimation and reveals the genetic architecture and molecular mechanisms of BAG by incorporating diverse functional genomic data from the human brain.

## Results

### Predicting brain age based on MRI with improved accuracy

**Figure 1:**
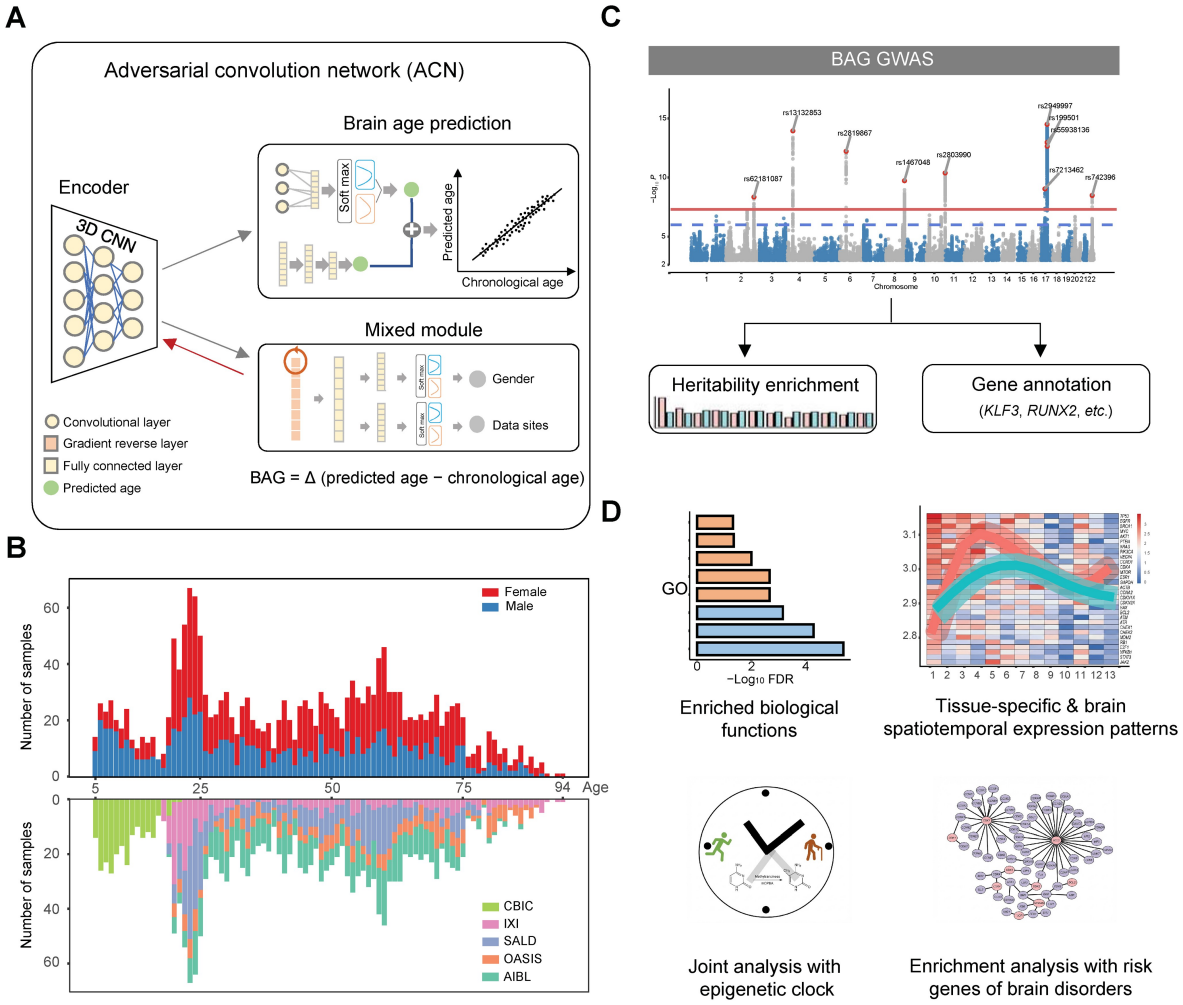
Workflow for brain age prediction, BAG GWAS, and downstream functional analyses **A**. The ACN framework for brain age prediction. **B**. Distribution of age and gender across participants from five independent datasets: CBIC, IXI, SALD, OASIS, and AIBL. **C**. BAG GWAS and post-GWAS analyses. **D**. Characterization of BAG-associated genes, including enriched biological functions (GO), expression dynamics, relationships with the epigenetic clock, and associations with brain disorders. BAG, brain age gap; GWAS, genome-wide association study; CBIC, Citigroup Biomedical Imaging Center; IXI, Information eXtraction from Images; SALD, Southwest University Adult Lifespan Dataset; OASIS, Open Access Series of Imaging Studies; AIBL, Australian Imaging Biomarkers and Lifestyle; ACN, adversarial convolution network; 3D CNN, three-dimensional convolutional neural network; GO, gene ontology; FDR, false discovery rate.

The heterogeneity of medical images often hampers the generalization capability of machine learning and deep learning models, posing challenges when applied to new datasets. To overcome these limitations, we developed an ACN (Figure S1) based on deep learning for accurately predicting brain age. Briefly, our model utilized a domain-adversarial training strategy [20], wherein we considered irrelevant factors (*e.g.*, gender and data sites) as unique domains in mixed modules and implemented dynamic restrictions on these modules during the training process (**Figure 1**A, Figure S1). To improve the generality and robustness of our model in predicting BAG, we performed model training and evaluation on the MRI datasets from 2011 healthy participants with ages ranging from 5 to 94 years, which were collected from 5 independent datasets (Figure 1B; Table S1), including the Southwest University Adult Lifespan Dataset (SALD) [21], Citigroup Biomedical Imaging Center (CBIC) [22], Australian Imaging Biomarkers and Lifestyle (AIBL) study of aging [23], Information eXtraction from Images (IXI; https://brain-development.org/ixi-dataset/), and Open Access Series of Imaging Studies (OASIS) [24]. We compared the performance of our ACN model with other models (**Table 1**, Table S2), including least absolute shrinkage and selection operator (LASSO) regression, elastic network regression, ridge regression, Gaussian process regression (GPR), support vector regression (SVR), three-dimensional residual network (3D ResNet) [14], and simple fully convolutional network (SFCN) [7], as well as ACN-based baseline models. We found that the ACN model showed superior performance over other models in terms of mean absolute error (MAE) (Table** 1**).

**Table 1:** Performance of ACN and other methods in predicting brain age on the test dataset

Model	MAE	*R* ^2^	PCC	Feature type
Ridge regression	6.02	0.88	0.94	GM and WM
GPR	6.03	0.88	0.94	GM and WM
LASSO regression	6.03	0.87	0.94	GM and WM
Elastic network regression	6.04	0.88	0.94	GM and WM
SVR	6.18	0.87	0.93	GM and WM
3D ResNet	5.06	0.83	0.95	T1-weighted
SFCN	5.01	0.89	0.95	T1-weighted
ACN baseline	4.09	0.88	0.91	T1-weighted
ACN baseline + gender	4.54	0.82	0.91	T1-weighted
ACN baseline + data site	4.87	0.87	0.91	T1-weighted
ACN	3.75	0.89	0.94	T1-weighted

*Note*: GPR, Gaussian process regression; LASSO, least absolute shrinkage and selection operator*;* SVR, support vector regression; 3D ResNet, three-dimensional residual network; SFCN, simple fully convolutional network; ACN baseline, a simplified ACN model only containing the brain age prediction module; ACN baseline + gender, adding a mixed module of gender to the ACN baseline model; ACN baseline + data site, adding a mixed module of data site to the ACN baseline model; ACN, adversarial convolution network; MAE, mean absolute error;$\;R^{2}$, coefficient of determination; PCC, Pearson correlation coefficient; GM, gray matter; WM, white matter; T1-weighted, T1-weighted MRI image; MRI, magnetic resonance imaging.

Next, we sought to validate the performance and robustness of ACN. We first explored the capability of ACN by restraining encoders from acquiring confounding factors, including gender and data sites. We extracted stratified features (*i.e.*, features after encoding) from the training data (Figure S1), and visualized their distributions with respect to gender and data sites using *t*-distributed stochastic neighbor embedding (*t*-SNE) (Figures S2 and S3). We found that ACN enhanced the encoder generalizability by effectively combining features from different genders and data sites. Second, we examined whether the stratified features were associated with brain aging. To this end, we used our pre-trained ACN model to extract the stratified features from 437 participants (aged 55–91 years, including 150 AD patients) from the Alzheimer’s Disease Neuroimaging Initiative (ADNI) [25], in which the individuals were diagnosed with a typical brain aging-related disorder (see Materials and methods). Subsequently, we utilized a support vector machine (SVM) classifier for diagnosing AD based on the stratified features. The SVM classifier achieved high accuracy (0.79 ± 0.28, 5-fold cross-validation) in distinguishing AD patients (Figure S4), suggesting that the stratified features are biologically meaningful and that the brain imaging features derived from AD patients are associated with brain aging [26].

### Identification of novel BAG-associated genetic variants

Knowledge of the genetic basis of BAG has markedly improved in recent years [13,27,28]. However, identifying novel genetic variants and genes associated with BAG in large-scale studies remains challenging. The UKB is an ideal resource for the genetic analysis of BAG, because it provides paired MRI and genotype data from a large cohort [29]. However, we observed an obvious age distribution difference between the UKB cohort (aged 45–85 years) and our training set (aged 5–94 years), which may bias the results of model prediction. To this end, we adopted a transfer learning approach to fine-tune the pre-trained ACN model and then applied it to the elderly cohort from the UKB. We further investigated the genetic basis of BAG (Figure 1C) and performed functional genomic analyses to elucidate the underlying regulatory mechanisms of BAG-associated variants and genes (Figure 1D).

**Figure 2:**
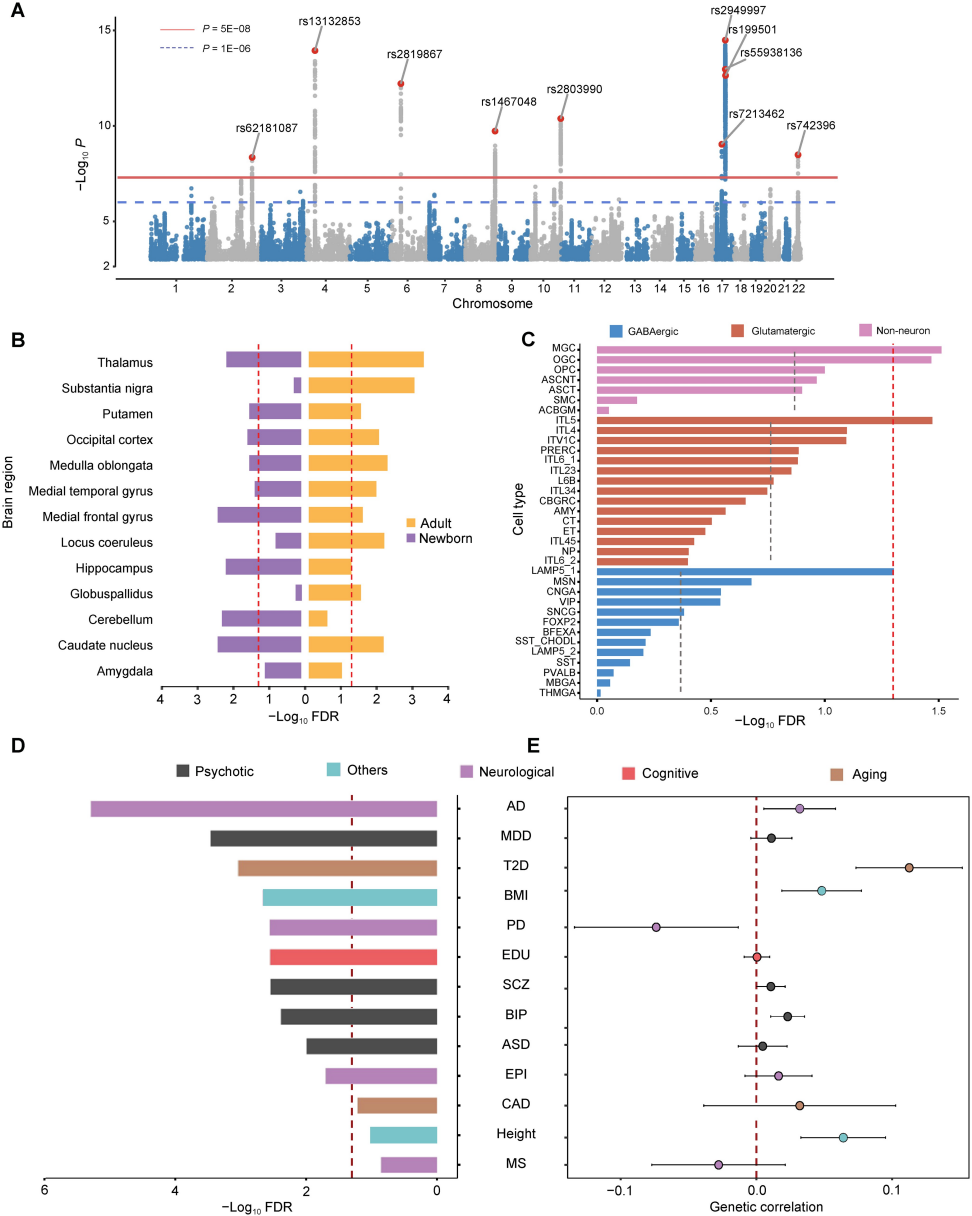
GWAS analysis of BAG **A**. Identification of novel BAG-associated genetic variants by BAG GWAS. The red line indicates the genome-wide significance threshold of *P* = 5E−08 in the GWAS summary statistics. The blue dashed line denotes the significance threshold of *P* = 5E−06. **B**. Partitioned heritability enrichment in active cCREs from distinct brain regions. The red dashed line denotes the FDR threshold of 0.05. **C**. Partitioned heritability enrichment in active cCREs from distinct neural cell types. The red dashed line denotes the FDR threshold of 0.05. The gray dashed lines denote the average FDR value within each group of cell types. The full names of the cell types are provided in Table S6. **D**. Associations between BAG and the PGSs of the 13 complex traits, estimated using a linear regression model. The red dashed line denotes the FDR threshold of 0.05. **E**. Genetic correlations between BAG and the 13 complex traits. The red dashed line corresponds to the genetic correlation of zero, *i.e.*, the genetic effects on the complex traits are independent of the BAG. Error bars represent the 95% CIs of the genetic correlation coefficients. AD, Alzheimer’s disease; ASD, autism spectrum disorder; BIP, bipolar disorder; BMI: body mass index; CAD, coronary artery disease; EDU, education; EPI: epilepsy; MDD, major depressive disorder; MS, multiple sclerosis; PD, Parkinson’s disease; SCZ, schizophrenia; T2D, type 2 diabetes; cCRE, candidate *cis*-regulatory element; FDR, false discovery rate; PGS, polygenic score; CI, confidence interval.

We performed a genome-wide association study (GWAS) of BAG using a larger sample size (*n* = 35,702) than previous studies [13,27,28,30]. Briefly, we estimated BAG for 35,702 participants using the ACN model (MAE = 2.70) (Figure S5), and then performed GWAS using Genome-wide Complex Trait Analysis (GCTA) [31] (see Materials and methods). In total, 3868 genetic variants that exceeded the genome-wide significance threshold (*P* < 5E−08) were identified (Table S3). Following linkage disequilibrium (LD)-based clumping (LD $r^{2}$ < 0.1), we identified ten lead variants, among which nine were novel and one (rs13132853) has been reported as the lead single nucleotide polymorphism (SNP) in a recent study [28] (**Figure 2**A, Figure S6; Table S4). In addition, five of the nine novel lead SNPs exceeded the genome-wide significance threshold (*P* < 5E−08) in the recent study [28] (Table S5).

Several lead SNPs identified in our study have been reported to be associated with brain structural features and blood cell morphology [32–34]. For example, rs13132853 and rs2819867 have been associated with radial diffusivity of white matter microstructure [33] and cortical thickness [32,34], respectively. We then evaluated the genome-wide genetic heritability ($h_{g}^{2}$) of BAG, and found that the genetic heritability of BAG from our GWAS [$h_{g}^{2}$ = 0.21, standard error (SE) = 0.02] was comparable to those from recent studies ($h_{g}^{2}$ = 0.18, SE = 0.02 from Kaufmann et al. [13]; $h_{g}^{2}=0.27,$ SE = 0.04 from Leonardsen et al. [28]). We also confirmed that our BAG GWAS summary statistics showed high genetic correlations with the results from existing studies ($r_{g}\;$= 0.56, *P* = 2.07E−20 for Kaufmann et al. [13]$;\;r_{g}$ = 0.82, *P* = 1.53E−59 for Leonardsen et al. [28]; Student’s *t*-test).

### Heritability of BAG is enriched in regulatory regions and implicated in glial cells

We performed partitioned LD score regression (LDSC) [35] to estimate the enrichment of genetic heritability of BAG in the putative functional genomic regions and in specific brain regions and cell types. As expected, we observed significant heritability enrichment in four genomic regions (Figure S7), with the strongest enrichment in super-enhancer regions [enrichment = 2.61, false discovery rate (FDR) = 2.97E−08, Student’s *t*-test] and enhancer-associated H3K27ac regions (enrichment = 1.87, FDR = 5.79E−05, Student’s *t*-test). These results reveal that the genome-wide genetic heritability of BAG is particularly enriched in the enhancer regions of human genome.

We then analyzed the enrichment of genetic signals from the BAG GWAS summary statistics within the candidate *cis*-regulatory elements (cCREs) from different brain regions and cell types using partitioned LDSC [35]. Given a broad range of neural samples from human brain available in the Functional Annotation of the Mammalian Genome 5 (FANTOM5) Project [36], we leveraged the annotated active cCREs (*i.e.*, promoters and enhancers) in 13 brain regions from early postnatal and adult periods in this analysis. We found that BAG showed significant heritability enrichment in active cCREs from diverse brain regions, especially in the adult (Figure 2B). Additionally, we investigated the heritability enrichment in the cCREs across 43 brain cell types (Table S6) [37], and found that BAG exhibited stronger heritability enrichment in glial cells than in neurons, which was consistent with previous studies showing that glial cells were more sensitive to brain aging and implicated in degenerative disorders such as AD and Parkinson’s disease (PD) [38–40] (Figure 2C).

### BAG is genetically associated with brain- and aging-related disorders

Previous studies have indicated that BAG shows variable genetic correlations with diverse brain disorders and traits [13]. However, the susceptibility and causal associations between BAG and brain disorders remain unclear. The polygenic score (PGS) is useful for estimating an individual’s genetic predisposition to a specific trait [41]. For each individual in the elderly cohort from UKB, we estimated the PGS for each of the 13 complex traits, including four neurological disorders [AD, epilepsy (EPI), multiple sclerosis (MS), and PD], four psychiatric disorders [autism spectrum disorder (ASD), major depressive disorder (MDD), BIP, and SCZ], two aging-related disorders [type 2 diabetes (T2D) and coronary artery disease (CAD)], one cognitive trait [education (EDU)], and two background traits [body mass index (BMI) and height] (Table S7). We then examined the associations between BAG and these PGSs across the cohort based on a regression model [42]. Our findings revealed significant positive associations between BAG and the PGSs for neurological and aging-related disorders, such as AD, PD, and T2D (FDR < 0.05, Student’s *t*-test) (Figure 2D), consistent with a previous study showing that these disorders could expedite the process of brain aging [43]. We also observed significant positive associations between BAG and the PGSs for psychiatric disorders, such as MDD, BIP, and SCZ (FDR < 0.05, Student’s *t*-test) (Figure 2D), consistent with the Enhancing Neuroimaging Genetics through Meta-analysis (ENIGMA) Consortium reports of elevated BAG in patients diagnosed with MDD and SCZ [44,45]. As expected, BAG displayed no significant association with the PGS for height (Figure 2D), used as the background trait here and assumed not to be implicated in brain aging.

Following the observation of robust associations between BAG and the PGSs for 10 of the 13 complex traits (Figure 2D), we next explored whether BAG shared its genetic architecture with these traits. We found that BAG showed positive genetic correlations with multiple brain-related and aging-related disorders, as well as the cognitive trait (Figure 2E). Notably, the genetic correlations between BAG and most brain-related disorders were generally weak ($r_{g}$ < 0.1) without reaching statistical significance, which was consistent with a previous study [13].

**Figure 3:**
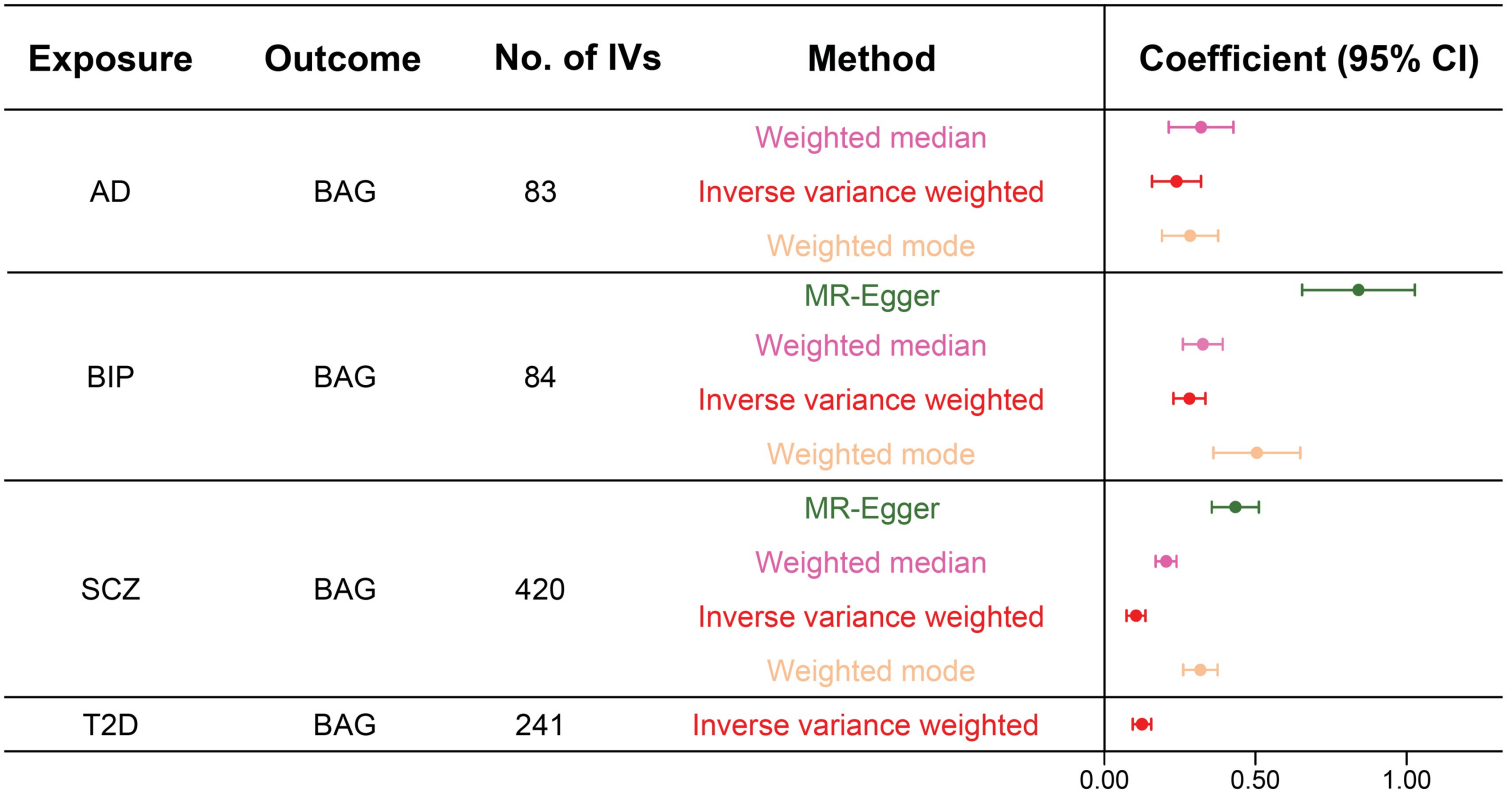
Genetic causal effects of complex traits on BAG Significant causal effects of the complex traits (exposure) on BAG (outcome) after adjusting for multiple testing using the Benjamini–Hochberg procedure to control the FDR threshold of 0.05. No. of IVs indicates the number of genetic variants used as instrumental variables. MR methods and their regression coefficients (causal effect estimates) are labeled with different colors. Error bars represent the 95% CIs of the regression coefficients. MR, Mendelian randomization.

In addition, we examined the underlying causal genetic associations between BAG and the complex traits using Mendelian randomization (MR) [46] (Tables S8 and S9). We identified significant causal genetic relationships (FDR < 0.05, *F* test) of one neurological disorder (AD), two psychiatric disorders (BIP and SCZ), and one aging-related disorder (T2D) with BAG (**Figure 3**, Figures S8 and S9; Tables S10 and S11). For example, the genetic causal effects of SCZ on BAG indicate that SCZ may cause pathogenic changes in the brain and further lead to accelerated brain aging, which has been supported by a previous study [10]. These results suggest that the accelerated BAG is likely a result, rather than a cause, of brain- and aging-related disorders.

### BAG-associated genes are enriched in aging-related biological pathways

To identify genes associated with BAG, we performed gene-based association analysis based on our BAG GWAS summary statistics data using Multi-marker Analysis of GenoMic Annotation (MAGMA) [47]. In this step, the genetic heritability of BAG was aggregated to the target genes by genomic proximity, where we identified 48 protein-coding genes (*P* < 2.64E−06, *F* test). To validate the reliability of the MAGMA-derived gene set, we also mapped the significant genetic variants (*P* < 5E−08 in the BAG GWAS summary statistics) to their target genes based on expression quantitative trait loci (eQTLs) and chromatin interactions from high-throughput chromosome conformation capture (Hi-C) data from human brain using FUMA [48], resulting in two additional sets of 64 and 78 genes, respectively [48]. These gene sets prioritized using functional genomic data showed significant overlap with the MAGMA-derived gene set (*P* < 2.2E−16 for eQTL and Hi-C, Fisher’s exact test) (Figure S10; Table S12). Some well-known BAG-associated genes, including *RUNX2*, *MAPT*, *ANKRD11*, and *KLF3*, were identified by both approaches [13,14,28].

**Figure 4:**
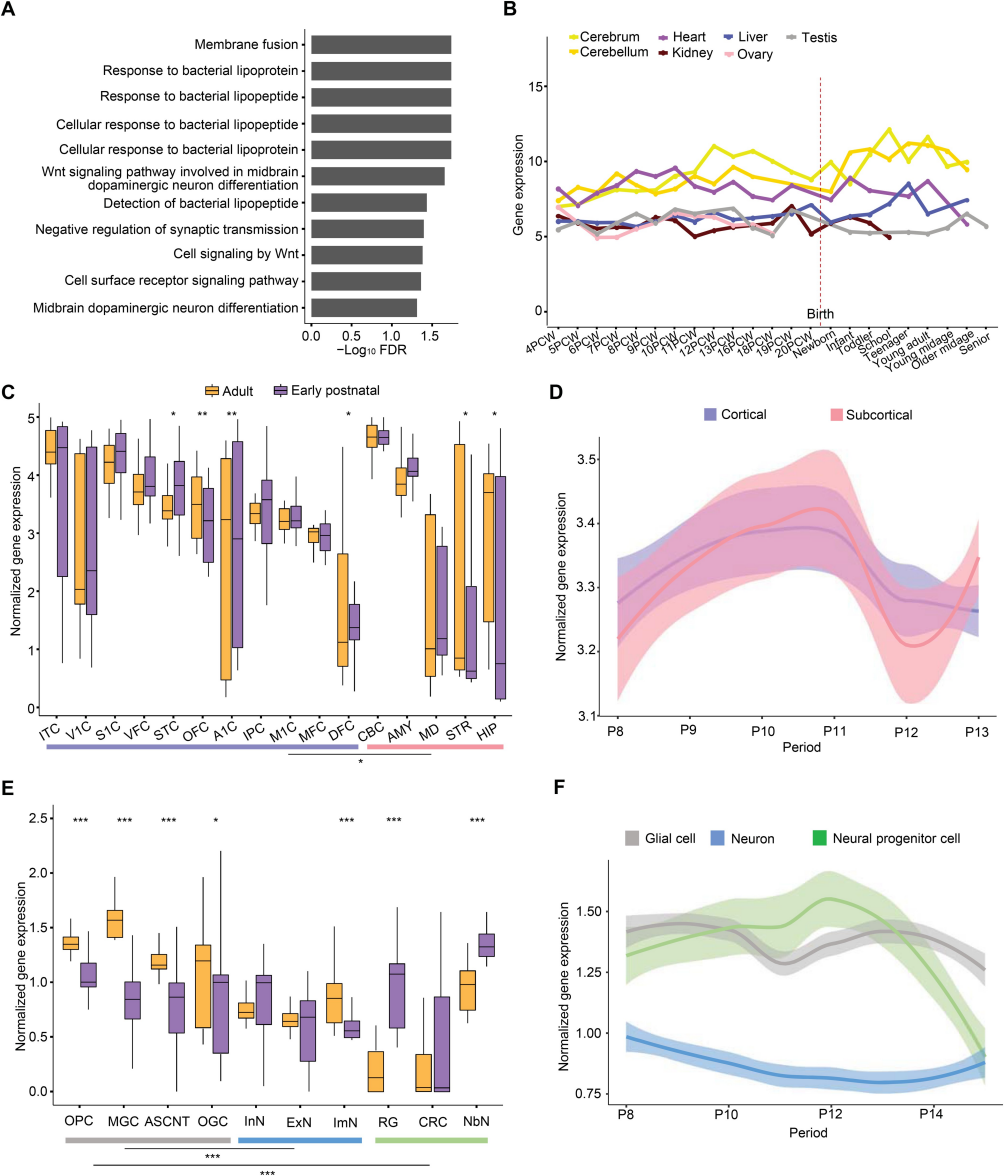
Enriched biological functions and expression dynamic patterns of BAG-associated genes **A**. Significantly enriched biological functions of BAG-associated genes (FDR < 0.05). **B**. Expression dynamics of BAG-associated genes in different tissues across lifespan. The expression data were obtained from Cardoso-Moreira and colleagues [54]. **C**. Expression of BAG-associated genes at adult *versus* early postnatal stages across different brain regions. The 16 brain regions are categorized as cortical (OFC, STC, VFC, MFC, IPC, DFC, S1C, V1C, ITC, M1C, and A1C) and subcortical (AMY, STR, MD, CBC, and HIP). *, *P* < 0.05; **, *P* < 0.01 (Wilcoxon test). **D**. Expression dynamics of BAG-associated genes in cortical and subcortical regions. The expression data were obtained from Zhu and colleagues [55]. **E**. Expression of BAG-associated genes at adult *versus* early postnatal stages in glial (gray), neuronal (blue), and neural progenitor (green) cells. *, *P* < 0.05; ***, *P* < 0.001 (Wilcoxon test). **F**. Expression dynamics of BAG-associated genes in glial, neuronal, and neural progenitor cells. The expression data were obtained from our STAB2 database [58]. In (D and F), the LOESS plots show smooth curves with 95% confidence bands. P8, birth ≤ age < 6 months; P9, 6 months ≤ age < 1 year; P10, 1 year ≤ age < 6 years; P11, 6 years ≤ age < 12 years; P12, 12 years ≤ age < 20 years; P13, 20 years ≤ age < 40 years; P14, 40 years ≤ age < 60 years. PCW, postconceptional week; OFC, orbital prefrontal cortex; STC, superior temporal cortex; VFC, ventrolateral prefrontal cortex; MFC, medial prefrontal cortex; IPC, inferior posterior parietal cortex; DFC, dorsolateral prefrontal cortex; S1C, primary somatosensory cortex; V1C, primary visual cortex; ITC, inferior temporal cortex; M1C, primary motor cortex; A1C, primary auditory cortex; AMY, amygdala; STR, striatum; MD, mediodorsal nucleus of thalamus; CBC, cerebellar cortex; HIP, hippocampus; OGC, oligodendrocyte; MGC, microglia; ASCNT, astrocyte; OPC, oligodendrocyte progenitor cell; InN, inhibitory neuron; ExN, excitatory neuron; ImN, immature neuron; RG, radial glia; CRC, Cajal-Retzius cell; NbN, newborn neuron; LOESS, locally estimated scatterplot smoothing.

We then performed gene set enrichment analysis to identify the enriched biological functions of these BAG-associated genes. We found that the BAG-associated genes were enriched in brain aging-related biological pathways (**Figure 4**), such as Wnt signaling pathway, which was critical for synaptic plasticity and maintenance in the adult brain [49]. Dysregulation of Wnt signaling pathway has been implicated in the functional decline during aging and pathogenesis of neurodegenerative diseases such as AD [50]. As expected, the BAG-associated genes were predominantly enriched for biological functions such as bacterial lipoprotein and synaptic transmission (Figure 4A). For example, bacterial lipoproteins have been identified as a key factor implicated in neuroinflammation and neurodegenerative diseases [51–53]. These enriched biological functions suggest that the BAG-associated genes we identified are biologically meaningful, and there is a substantial association between these genes and the process of brain aging.

### BAG-associated genes show elevated expression in adult brain regions and glial cells

Hypothesizing that the BAG-associated genes are functional in regulating aging in brain and/or whole body, we investigated the expression dynamics of the BAG-associated genes using transcriptomic datasets from seven different tissue types (including cerebrum, cerebellum, and five other non-brain tissue types) across human lifespan [54]. The BAG-associated genes exhibited consistently higher expression levels in the cerebrum and cerebellum compared to non-brain tissue types throughout the majority of the lifespan (Figure 4B). Although BAG can represent the process of aging in the whole body, these results suggest that the BAG-associated genes are relatively more associated with human brain and may be functional in human brain.

Next, we systematically compared the expression levels of BAG-associated genes between the early postnatal and adult periods across 16 brain regions [55]. We found that BAG-associated genes showed significantly higher expression in the adult period than in the early postnatal period in four brain regions, including primary auditory cortex, orbital prefrontal cortex, striatum, and hippocampus (*P* < 0.05, Wilcoxon test) (Figure 4C), which are related to the functions of perception and memory [56,57]. We also noticed that BAG-associated genes generally had significantly higher expression in cortical regions than in subcortical regions during aging (*P* = 0.03, Wilcoxon test) (Figure 4C). Given these observations, we then characterized the expression dynamics of BAG-associated genes in cortical and subcortical regions from birth to adulthood. The cortical regions showed an earlier expression elevation of BAG-associated genes than the subcortical regions from the beginning of adulthood (Figure 4D), suggesting that the cortical regions may age earlier than the subcortical regions [2].

We further explored the expression patterns of BAG-associated genes in brain cell types, utilizing the single-cell RNA sequencing data from the STAB2 database [58]. Consistent with our previous results from partitioned heritability enrichment in brain cell types, BAG-associated genes were significantly highly expressed in adult brain cells (*P* < 2.2E−16, Wilcoxon test), particularly in glial cells such as oligodendrocytes and microglia (Figure 4E). In addition, during aging, BAG-associated genes showed consistently higher expression levels in glial cells than in neurons (Figure 4F). These observations confirmed the results mentioned above that glial cells were more sensitive to the aging process.

### BAG-associated genes exhibit strong functional correlations with aging-related epigenetic clock

**Figure 5:**
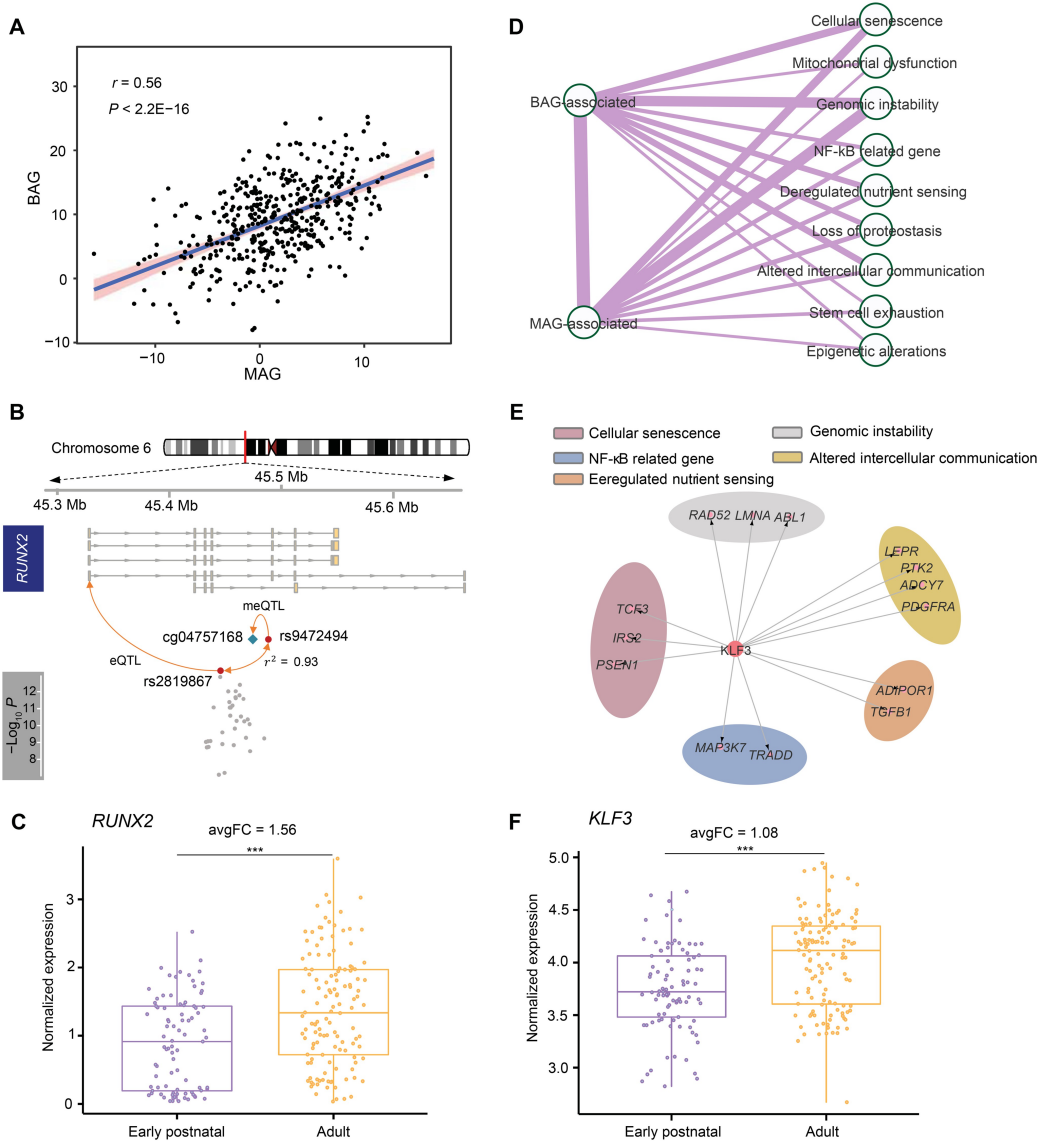
Genetic association between BAG and MAG **A**. Association between BAG and MAG in the ADNI dataset. **B**. Shared genetic loci between BAG and the epigenetic clock in *RUNX2*. **C**. Expression of *RUNX2* in brain regions at early postnatal *versus* adult stages. ***, *P* < 0.001 (Wilcoxon test). **D**. Overlap between BAG/MAG-associated genes and different aging-related biological pathways. The line width indicates the correlation strength among different gene sets. **E**. An example showing the target genes regulated by *KLF3* in different aging-related biological pathways. **F**. Expression of *KLF3* in brain regions at early postnatal *versus* adult stages. ***, *P* < 0.001 (Wilcoxon test). MAG, methylation age gap; ADNI, Alzheimer’s Disease Neuroimaging Initiative; eQTL, expression quantitative trait locus; meQTL, DNA methylation quantitative trait locus; avgFC, averaged fold change.

It is crucial to interpret the underlying associations between BAG and established aging-related biological processes. However, there have been few studies aiming to explicate their connections by utilizing BAG as a marker and assessing its correlations with cognitive phenotypes [59]. Here, we established a link between brain aging and biological aging through the regulatory mechanism of epigenetic clock, defined as a specific collection of CpG sites whose DNA methylation levels can be used to estimate a subject’s age [60]. The methylation age gap (MAG), defined as the difference between the predicted biological age based on methylation signals in blood and the chronological age, has been widely used as a biomarker of aging [61]. Briefly, we predicted the methylation age and brain age for the ADNI cohort using a predefined epigenetic clock model [62] and our ACN model, respectively, followed by calculating the MAG and BAG for each individual. The BAG and MAG showed a concordant pattern in patients diagnosed with AD, who had significantly higher BAG than healthy participants (Figure S11). We observed a significant association between BAG and MAG (Pearson correlation coefficient *r* = 0.56, *P* < 2.2E−16) (**Figure 5**A), indicating that our predicted brain age is reliable to represent the chronological age and can be used to characterize the potential aging processes. Furthermore, we identified a set of MAG-associated genes based on the epigenetic clock and evaluated their functional similarity to BAG-associated genes. We observed a high degree of functional similarity between these two gene sets (GOSim = 0.80 [63]).

Here, we exemplified *RUNX2*, a gene identified in our analysis as associated with both BAG and MAG, to show the complex regulatory relationships between BAG and the epigenetic clock (Figure 5B). *RUNX2* has been identified as the regulatory target of the eQTL SNP rs2819867 [64], which was also identified as the BAG lead SNP in our study and in strong LD with a nearby SNP rs9472494 ($r^{2}$ = 0.93). Interestingly, the SNP rs9472494 has been identified as a DNA methylation quantitative trait locus (meQTL) for cg04757168 [65], which is located within *RUNX2*. RUNX2 has been reported to be implicated in the pathogenesis of aging-related disorders, such as PD [66]. Consistent with these findings, *RUNX2* showed elevated expression in the adult brain than in the early postnatal brain [fold change (FC) = 1.56, *P* = 7.6E−06, Wilcoxon test] (Figure 5C).

As we found that BAG-associated genes showed significant functional overlap with MAG-associated genes and could be linked to aging processes, we raised the question of which aging-related biological pathways were implicated by both BAG-associated and MAG-associated genes. To this end, we first collected aging-related gene sets, which were organized into 10 categories of aging-related biological functions or pathways [67]. We then examined the potential regulatory relationships between BAG-associated genes, MAG-associated genes, and aging-related biological functions or pathways separately in the transcriptional regulatory networks (TRNs) [68]. BAG-associated genes showed higher connectivity with MAG-associated genes in the TRNs compared to other genes [mean connectivity score ($C_{s}$) = 1.64] (Figure S12). Both BAG-associated and MAG-associated genes were highly implicated in the aging-related biological functions or pathways, particularly in genomic instability and cellular senescence (Figure 5D). Intriguingly, *KLF3*, a key TF gene in the BAG-associated genes, was identified as the regulator of numerous aging-related genes, particularly those involved in intercellular communication (*e.g.*, *LEPR*, *PTK2*, and *ADCY7*) and cell senescence (*e.g.*, *IRS2* and *PSEN1*) (Figure 5E), which have been implicated in synapse formation, lipid metabolism, and insulin resistance [69,70]. Additionally, *KLF3* showed significantly higher expression in the adult brain (FC = 1.08, *P* = 3.4E−06, Wilcoxon test) (Figure 5F), and regulated 23 MAG-associated genes, which were involved in regulating immune-related functions and cell development [71–74].

Taken together, these findings suggest that the aging processes estimated by epigenetic signals in blood share certain biological functions and/or pathways with brain aging, particularly in the functional categories of immune senescence and cell senescence-associated pathways.

### Genetic pleiotropy of BAG-associated genes in brain disorders

The aging trajectory of the brain has been implicated in brain disorders [2]. Nonetheless, the functional mechanisms bridging brain aging and brain disorders remain to be comprehensively understood. We thus investigated the potential biological associations between brain aging and neurological disorders, by utilizing molecular network analysis to capture the associations between BAG-associated genes and seven different brain disorders [75] (Table S13).

**Figure 6:**
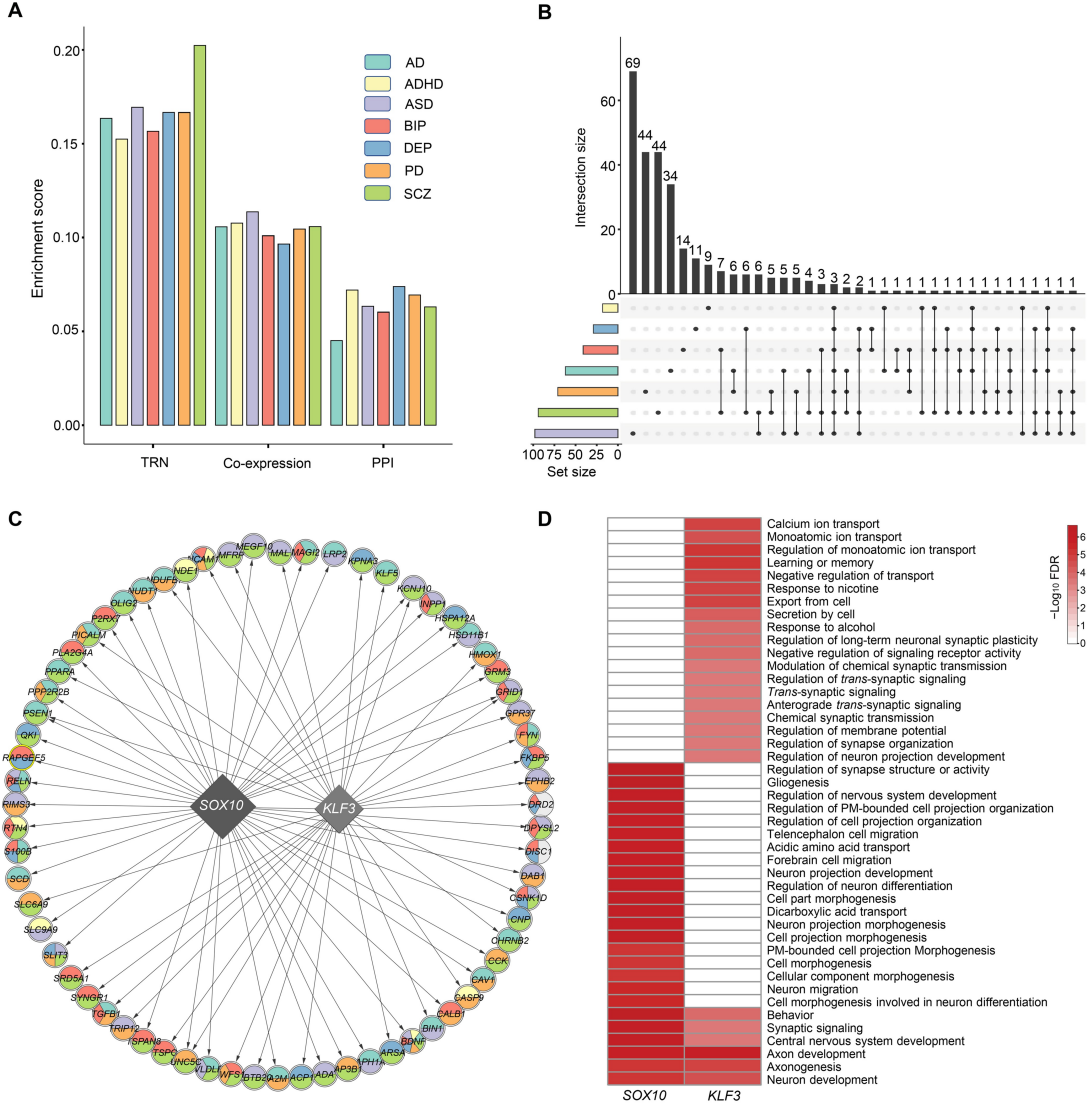
Pleiotropy between BAG-associated genes and risk genes for brain disorders in molecular networks **A**. Enrichment of BAG-associated genes with risk genes for diverse brain disorders in molecular networks. Enrichment was estimated using the Jaccard distance index, with higher values indicating greater similarity. **B**. Overlap of the risk genes that interact with BAG-associated genes within brain TRNs across multiple brain disorders. **C**. Delineation of pivotal hub modules centered on two BAG-associated genes (*KLF3* and *SOX10*; diamonds), which are linked to risk genes for multiple brain disorders (circles). Each risk gene is annotated with a pie chart color-coded by its associated brain disorders. **D**. Significantly enriched biological functions of the target genes regulated by BAG-associated hub TF genes, *KLF3* and *SOX10*. ADHD, attention-deficit/hyperactivity disorder; DEP, depression; TRN, transcriptional regulatory network; PPI, protein–protein interaction; TF, transcription factor.

We examined three types of molecular networks in the human brain to uncover the potential associations of BAG-associated genes with brain disorders, including TRNs, protein–protein interaction (PPI) networks, and gene co-expression networks. Our results highlighted that BAG-associated genes and many risk genes for neurological disorders were more strongly enriched in the TRNs than in the gene co-expression and PPI networks (**Figure 6**A, Figures S13–S15). These results suggest that the TRNs in the human brain may better represent the potential regulatory relationships underlying brain aging and disorders [76].

To explore the impact of brain aging on diverse brain disorders, we examined the pleiotropy of disorder-associated risk genes modulated by BAG-associated genes within the brain TRNs. Our results indicated that 22.68% of the risk genes that had interactions with BAG-associated genes were involved in at least two brain disorders (Figure 6B). Intriguingly, we detected two major hub modules (ranked by degree) regulated by *KLF3* and *SOX10*, whose target genes were enriched for risk genes for diverse brain disorders (Figure 6C). The risk genes regulated by *KLF3* and *SOX10* were significantly enriched for biological functions in brain development (*e.g.*, central nervous system development and neuron development) and cognition (*e.g.*, learning or memory) (Figure 6D).

We also explored the interactions between BAG-associated genes and risk genes for diverse disorders within the co-expression and PPI networks in the human brain. Consistent with the results from the brain TRNs, 34.10% and 50.0% of the risk genes that had interactions with BAG-associated genes were associated with at least two brain disorders in the co-expression and PPI networks from the human brain, respectively (Figures S16 and S17).

Given that our MR results revealed causal effects of psychiatric and neurological disorders on BAG, we also examined the regulatory influence of their risk genes on BAG-associated genes. As expected, we found that the BAG-associated gene *MAPT* was regulated by several risk genes for psychiatric disorders, such as *CLOCK* and *ARNTL* [77]. *MAPT* has been reported as functional in regulating cognition [78]. These results suggest that certain psychiatric and neurological conditions may accelerate brain aging by affecting the underlying mechanisms of cognition [79,80].

## Discussion

Our study presents a catalog of BAG-associated genetic variants and genes, identified by the ACN model, a novel computational framework for brain age prediction. With this catalog, we investigate the expression patterns and regulatory mechanisms of the BAG-associated genes and uncover their associations with the epigenetic clock and aging-related biological pathways.

Previous studies suggest that the aging process of the brain exhibits gender differences, with female brains generally younger than male brains [81,82]. Although gender has been well considered as a confounding factor in predicting brain age in most established computational models, it should also be noted that there exist indeed sex-biased differences in brain structure [30], which are usually ignored in most current models. To address this challenge, we proposed ACN, an adversarial model to minimize the influence of sex-biased differences of brain structure in brain age prediction. The main advantage of our ACN model is that we utilized a domain-adversarial training strategy to reduce the influence of noise and gender effects from different data sites, and make the stratified features more relevant to the real brain age (Figure S18). We found that this approach could improve the accuracy of brain age prediction compared to alternative models, laying a solid foundation for the downstream GWAS and prioritization of BAG-associated genes.

We estimated the genetic correlations and causal relationships between BAG and multiple brain/aging-related disorders. As expected, BAG exhibited complex genetic associations with these disorders rather than with background traits, such as height, suggesting that the BAG estimation by our ACN model was biologically meaningful and could reflect brain- and age-related physiological changes. Notably, our results suggest that neurological (AD), psychiatric (SCZ and BIP), and aging-related (T2D) disorders could induce accelerated brain aging. We identified nine novel BAG-associated genetic variants. Furthermore, we prioritized 44 novel genes associated with BAG and showed that these BAG-associated genes had significantly elevated expression in brain aging-related regions and cell types (*e.g.*, adult hippocampus and glial cells). Some BAG-associated genes (*e.g.*, *RUNX2*) showed genetic overlap with the epigenetic clock and aging-related biological pathways (*e.g.*, cell senescence). Finally, we examined the potential regulatory relationships between BAG-associated genes and risk genes for brain disorders in molecular networks. We highlighted two pleiotropic BAG-associated TF genes (*KLF3* and *SOX10*) that potentially play key regulatory roles in different brain disorders.

Brain aging is a complex biological process, and BAG, derived from MRI data, can be used as a reliable measure for quantitatively assessing this process. However, BAG is typically predicted based on computational models using different brain imaging data, and its relevance to the biological processes of aging remains unclear. Great efforts have been made to determine whether the predicted BAG is physiologically reliable by examining its relations with brain-associated phenotypes, such as cognitive ability or mental disorders. Over the past decade, epigenetic clock has emerged as a highly accurate molecular indicator of biological age in humans [83]. In this study, we proposed to validate BAG as a novel indicator of brain aging by revealing the putative connection between BAG and biological aging through the epigenetic clock. Our study showed that BAG-associated genes have strong correlations and functional overlaps with the genes involved in the epigenetic clock.

We revealed that the genetic heritability of BAG was significantly enriched in functional regulatory regions and glial cells. Glial cells play critical roles in regulating molecular processes including neuroinflammation and synaptic pruning. Dysregulation of these processes has been implicated in neurodegenerative diseases and aging-associated cognitive decline [84–86]. Our results suggest that accelerated brain aging has genetic associations with certain neurological and psychiatric disorders, such as AD and SCZ, both of which have been linked to the dysfunction of glial cells [87,88]. Taken together, these findings indicate that glial cells may play key roles in regulating brain aging, and the dysfunction of glial cells could induce diverse neurological and psychiatric disorders.

Previous studies have revealed that brain disorders can expedite brain aging and have uncovered significant independent loci associated with BAG (*e.g.*, rs2106786 and rs2790102) [13,28]. However, the associations between BAG, brain aging, and brain disorders at the gene level have not been well characterized. In our study, we validated well-known genes and identified novel genes that were associated with BAG. We further examined the interactions of BAG-associated genes with the risk genes for diverse brain disorders in biological networks, and found two common gene modules (*KLF3* and *SOX10*). *KLF3* has been associated with various aging-related biological pathways and can regulate numerous risk genes for diverse brain disorders, indicating its strong pleiotropy in brain disorders. The target genes of *KLF3* (*e.g.*, *TGFB1*, *FYN*, and *DRD2*) have been shown to participate in the regulatory processes of neurodevelopment and inflammation [89–93]. *SOX10* can significantly interact with some risk genes for neuroinflammation implicated in neurodegenerative disorders, such as *RNF11* and *LAMP1* [94,95] (Figure S17). Interestingly, we also found that BAG-associated genes *KLF3*, *MAPT*, *WNT3*, and *RUNX2* were all associated with the Wnt signaling pathway, which plays critical roles in the regulation of brain development, the aging process, and a variety of neurological disorders [96,97]. These results suggest that neuroinflammation and the Wnt signaling pathway are implicated in abnormal aging processes of the human brain and may be involved in the pathogenesis of psychiatric and neurodegenerative disorders [98–100]. Our previous results have shown that the BAG-associated variants and genes are significantly enriched in glial cells, which are closely related to neuroinflammatory processes [40].

The BAG metric used in this study was developed from structural MRI data collected from a large cross-sectional dataset of individuals spanning a wide age range, and it encapsulates the prediction error from a deep learning model. Therefore, its limitations include both noise (*i.e.*, model accuracy deficiencies and inadequate data quality) and physiology (*i.e.*, deviations from standard aging trajectories). Despite considering site effects and healthy conditions, caution is necessary when estimating the aging trajectories of different cohorts with limited population sizes. Given that the genetic architecture of BAG may be multifarious and population-specific, we only used data from individuals of European ancestry. Future studies may need to be repeated in other races as well as in larger cohorts. Furthermore, while previous studies have suggested that BAG can serve as a biomarker for characterizing the brain aging process, it may exhibit a stronger correlation with structural brain changes. In future work, the aging trajectory of the brain could be more comprehensively depicted by integrating various brain functional modalities with multi-omics data.

In conclusion, we investigated the genetic architecture of BAG, prioritized the BAG-associated genes, and revealed their biological significance and relationships to brain disorders at the functional genomic level. Our findings suggest that the BAG can serve as an effective biomarker for characterizing the processes of brain aging and related brain disorders.

## Materials and methods

### Collection and preprocessing of structural MRI data

We compiled a dataset of T1-weighted MRI images from five independent data sites: SALD [21], CBIC [22], AIBL [23], IXI, and OASIS [24] (Table S1). In total, this compiled dataset included 2011 healthy individuals (aged 5–94 years) and was used for model training and testing. During model training and testing, the MRI images were split into 70% (*n* = 1407) for training, 10% (*n* = 201) for validation, and 20% (*n* = 403) for testing.

We also leveraged two additional MRI datasets for model application: the UKB dataset (*n* = 38,702; aged 45–85 years) and the ADNI dataset (*n* = 437; aged 55–91 years). The UKB and ADNI datasets were used in the GWAS analysis of BAG, as well as in the association analysis between BAG and the epigenetic clock of aging.

We performed a five-step preprocessing pipeline for the raw MRI data: (1) N4 bias correction [101]; (2) skull stripping, which involves skull removal and brain extraction using FMRIB Software Library; (3) nonlinear registration of the images to the standard Montreal Neurological Institute (MNI) space using Advanced Normalization Tools (ANTs) [102]; (4) voxel-value normalization, with all voxel sizes aligned to 1.5 × 1.5 × 1.5 mm^3^; and (5) segmentation of the images into gray matter, white matter, and cerebral spinal fluid. Following preprocessing, the voxel resolution was 121 × 145 × 121.

### Development of ACN model for brain age prediction

We developed ACN, a novel computational model to predict brain age. This model comprises an encoder, a task classification (regression) module, and mixed modules. The encoder selectively extracts age-related features while protecting the feature domain from confounding variables such as data site and gender. Details of brain age prediction are shown in Figure S1.

In this study, we proposed a model that can integrate irrelevant factor mixed blocks by harnessing the principles of adversarial learning. These mixed-factor tasks hold a negative correlation with the principal task of brain age prediction. To achieve the brain age prediction (baseline model), two approaches were utilized. Initially, we used conventional regression with L1 loss to constrain the output. However, due to the heterogeneous distribution of age, a distribution constraint was also included. For each age label *y*, the cumulative distribution function of a normal distribution was employed to convert this into a probability distribution, resulting in a 90-dimensional vector (5–94). The conversion function is illustrated below:


(1) $$F(y;\mu ,\sigma )=\frac{1}{\sigma \sqrt{2\pi }}\int_{-\infty }^{y}\;\text{exp}\;\left(-\frac{{\left(y-\mu \right)}^{2}}{2\sigma^{2}}\right)dy$$

where μ and $\sigma^{2}$ are the expectation and variance for the age labels, respectively.

The output layer comprises 90 numbers representing the predicted probability that the subject’s age falls within a one-year age interval from 5 to 94. The Kullback–Leibler divergence computes the correlation for the probability distribution of the label and prediction as the loss. Consequently, the loss of brain age prediction can be formulated as follows:


(2) $$L_{\mathrm{age}}=\sum_{i=1}^{n}\left(|y_{i}-\bar{y_{i}}|+\sum_{j=1}^{90}\left[p_{y_{i}}(x_{j})\log p_{y_{i}}(x_{j})-p_{y_{i}}(x_{j})\log q_{{\bar{y}}_{i}}(x_{j})\right]\right)$$

where y is the true label, and $\bar{y}$ denotes the predicted value of the model. To validate the effectiveness of the two loss functions, we compared the performance of models trained with different loss constraints (Table S2).

In contrast, the mixed module might incorporate tasks that potentially affect the generalization of the main task, such as gender and different datasets. To hinder the encoder from extracting features associated with these factors and to align the domains based on gender and data site, we extended the domain adaptation method to the mixed module. Specifically, a gradient inversion layer was placed ahead of the mixed block to constrain the feature encoder. The gradient update strategy followed the same approach as used in the domain-adversarial neural networks (DANN) [20]. In this case, the loss for confusion factors was cross-entropy, which can be expressed as follows:


(3) $$L_{c}=\sum_{i=1}^{2}-\frac{1}{N}\sum_{j=1}^{N}\sum_{c=1}^{C_{i}}d_{c}^{\left(j\right)}\;\text{log}\;{\bar{d}}_{c}^{\left(j\right)}$$

where i is the number of different factors, $C_{i}\;$denotes the dimension for different classification model output, and $\bar{d}$ denotes the output of the classification model. The learning process minimizes the loss function that is written as follows:


(4) $$L_{\mathrm{all}}=\mathrm{argmin}(L_{\mathrm{age}}+L_{c})$$

To evaluate the effectiveness of the model featuring a mixed-factor module of gender and data site, we compared its results with those of a model without this module (Table 1).

The loss function was optimized by Adaptive moment estimation (Adam) [103] with a learning rate of 1E−04 and a batch size of 12. The training process consisted of 200 epochs, with each epoch including a training step and a validation step. Our model implementation utilized approximately 20 GB of memory. The training time took approximately 8 h, employing an AMD EPYC 7763 64-core processor with 2 TB of Random Access Memory (RAM) and an NVIDIA Tesla A100 80 GB Graphics Processing Unit (GPU).

### Applying the pre-trained ACN model to the UKB cohort

We adapted our pre-trained ACN model to the elderly cohort from the UKB through transfer learning. We first selected approximately 10% (*n* = 3000) of the individuals from the UKB cohort for model fine-tuning. These individuals were divided into three subsets: 70% for training, 10% for validation, and 20% for testing. The model was trained on the selected individuals (*n* = 3000) and fine-tuned over 100 epochs. The fine-tuned model showed improved performance on the test set, achieving an MAE of 2.51 and a Pearson correlation coefficient of 0.91. We then applied this optimized ACN model to the remaining individuals (*n* = 35,702) from the UKB cohort to predict their brain ages, followed by computing their BAG scores.

### GWAS summary statistics data of complex traits

We collected GWAS summary statistics for 13 complex traits (Table S7). Most of these GWAS summary statistics datasets were recently published and available, and were based on meta-analyses using extensive sample sizes of European ancestry. The sample size for each of the GWAS summary statistics datasets was as follows: (1) Neurological disorders: AD (428 cases and 429,961 controls) [104], EPI (15,212 cases and 29,677 controls) [105], MS (47,429 cases and 68,374 controls) [106], and PD (33,674 cases and 449,056 controls) [107]; (2) Psychiatric disorders: ASD (18,381 cases and 27,969 controls) [108], BIP (41,917 cases and 371,549 controls) [109], MDD (135,458 cases and 344,901 controls) [110], and SCZ (76,755 cases and 243,649 controls) [111]; (3) Cognitive traits: EDU (766,345 individuals) [112]; (4) Aging-related disorders: CAD (22,233 cases and 64,762 controls) [113] and T2D (34,840 cases and 114,981 controls) [114]; and (5) Background traits: BMI (694,649 individuals) [115] and height (450,000 individuals) [116].

### Genetic data preprocessing and association analysis

Genetic analyses were performed on individuals of European ancestry from the UKB, who also had genotype and T1-weighted MRI data. Standard quality control procedures were then applied to the UKB v3 imputed genetic data [117]. These procedures included the following steps: (1) exclusion of individuals with failed genotyping, abnormal heterozygosity status, or withdrawn consents; (2) removal of participants genetically related to another participant up to the third degree, as inferred by kinship coefficients implemented in PLINK [118]; (3) elimination of variants with a minor allele frequency below 0.01; (4) removal of variants with a genotype missing rate exceeding 10%; (5) exclusion of variants failing the Hardy–Weinberg equilibrium test at the 1E−07 level; and (6) elimination of variants with an imputation INFO score below 0.8. Post quality control, we retained 38,702 individuals and 9,055,103 variants.

In terms of the GWAS, we performed the BAG GWAS analysis using GCTA based on the generalized linear mixed model [119], with adjustments for some covariates, including age (at imaging), age squared, sex, sites, age–sex interaction, age squared–sex interaction, intracranial volume, imaging center, and the top 20 genetic principal components provided by UKB (Data-Field 22009). The *P* value threshold for selecting the significant GWAS tag variants is 5E−08.

We leveraged partitioned LDSC [35] to estimate the heritability enrichment in the regulatory elements active in each brain cell type and brain region. Briefly, LDSC regresses GWAS χ^2^ statistics on SNPs’ LD scores, reflecting the degree to which each SNP is correlated with its surrounding SNPs [120]. The LD score regression intercept of BAG GWAS was close to 1 (intercept = 0.97, SE = 6.8E−03), indicating the absence of genomic inflation of test statistics due to confounding factors. Moreover, the pre-calculated genome-wide LD scores were obtained from LDSC (https://alkesgroup.broadinstitute.org/LDSCORE/). The LD scores were calculated based on data from individuals of European ancestry from the 1000 Genomes Project [121]. We further removed SNPs which were not annotated in HapMap3 [122] or located in the major histocompatibility complex regions.

### PGS computation and correlation analysis with BAG

A PGS represents an estimate of an individual’s genetic predisposition for a given trait. For each individual in the UKB cohort, we computed the PGS for each trait based on the genotype data and the GWAS summary statistics dataset using PRSice-2 [123]. Notably, we double-checked the sample metadata of the collected GWAS summary statistics to ensure that there was no sample overlap between the UKB cohort and the GWAS cohorts used in our study (Table S7).

Prior to PGS computation, we performed quality control on the UKB genotype data, *e.g.*, removing duplicate and ambiguous SNPs, clumping to account for LD, pruning highly correlated SNPs, and filtering out high-heterozygosity samples. We applied the *P* value thresholding approach for PGS calculation using PRSice-2 [123], with *P* value thresholds ranging from 5E−08 to 1. We then performed linear regression analysis to examine the correlation between PGS and BAG while adjusting for several covariates, including age, sex, intracranial volume, and the top 10 genetic principal components. The optimal threshold for each trait was chosen by maximizing the incremental *R*^2^ in the linear regression model. All *P* values were converted into FDR by conducting multiple testing correction.

### MR analysis

We confirmed that there was no sample overlap between the UKB cohort and the GWAS cohorts used in the MR analysis. We then preprocessed the GWAS summary statistics datasets in accordance with standard MR preprocessing procedures. In the exposure GWAS, the genetic variants were selected based on a genome-wide significance threshold of 5E−08. To ensure the independence of genetic variants included in the subsequent MR analysis, we applied LD-based clumping with an $r^{2}$ threshold of 0.1 and a window size of 250 kb, and utilized the European ancestry data from the 1000 Genomes Project as the reference panel [121]. The MR procedure was performed using the R package TwoSampleMR (v0.4.26; https://mrcieu.github.io/TwoSampleMR/).

We also performed sensitivity analysis on the MR results. We used the harmonise_data function in TwoSampleMR to harmonize the effect alleles and SNP effects between the exposures and outcomes. We used the mr_heterogeneity function (*P* < 0.05) and the mr_pleiotropy_test function (*P* < 0.05) to detect the heterogeneity (Table S13) and horizontal pleiotropy (Table S14) in the MR results, respectively. We also used the mr_leaveoneout function to test the sensitivity of the MR results to individual instrumental variables.

### Prioritization of BAG-associated genes

In the BAG GWAS, a gene-based association analysis for 18,796 protein-coding genes was performed using MAGMA (v1.08) [47] with default parameter settings, including a zero-window size around each gene. Subsequently, functional mapping and annotation analyses were performed using FUMA [48], which involved annotating variants for biological functions and linking them to candidate target genes through an integration of eQTL data and chromatin interactions from Hi-C data in human brain. Brain-related tissues/cells were selected for all annotations using default parameters [48]. We used ToppGene [124] to identify the significantly enriched (FDR < 0.05) Gene Ontology terms of the BAG-associated genes.

### Collection and analysis of methylation data

We obtained the DNA methylation data from the ADNI cohort [125], which were generated using Illumina EPIC chips. Subsequently, we used the Chip Analysis Methylation Pipeline (CHAMP) to preprocess the methylation data with default parameters [126]. These steps included: (1) filtering out low-quality probes; (2) quality control of samples; (3) signal normalization; and (4) removing batch effects and regressing sex. After these steps, the remaining samples had an average CpG call of 695,127. Finally, we selected 437 individuals with brain MRI data for biological age prediction.

We applied a recently published elastic network model to predict the biological age based on the blood methylation data [62]. This model was trained on 13,402 samples with blood methylation data, identifying 514 CpGs as the epigenetic clock. For this study, the epigenetic clock was extracted as a feature, and the pre-trained elastic network model was used to predict the methylation age for each participant. Additionally, the epigenetic clock was mapped to 351 associated genes using the Illumina EPIC array annotation file.

### Collection of risk genes for brain disorders

Risk genes for brain disorders were compiled from various resources: (1)risk genes for attention-deficit/hyperactivity disorder (ADHD) were downloaded from the ADHDgene database (http://adhd.psych.ac.cn), selecting only those with support from at least 60% of all studies included in the database [127]; (2) risk genes for ASD were downloaded from the AutDB database (http://autism.mindspec.org/autdb), supplemented by recent studies [128,129]; (3) risk genes for SCZ were obtained from the SZGene database (http://szdb.org), supplemented by recent studies [64,130]; (4) risk genes for BIP were downloaded from DisGeNET [131]; (5) risk genes for MDD were downloaded from the Polygenic Pathways database [132]; (6) risk genes for AD were obtained from the ALzGene database (https://www.alzforum.org/alzgene) [133]; and (7) risk genes for PD were obtained from [134]. The full list of these risk genes is provided in Table S13.

### Collection of molecular networks

Three extensive molecular networks were utilized in this study to investigate the associations between BAG and brain disorders: (1) brain-specific TRNs were obtained from Pearl et al. [68], which included 741 TFs and 11,092 target genes; (2) brain-active PPI networks were reconstructed by obtaining the global PPI networks from the STRING database (https://string-db.org), followed by retaining protein pairs with physical interaction scores over 700 and proteins active in the adult human brain (expression value > 0) [55], which finally included 8568 proteins and 114,892 interaction edges (Table S14); (3) gene co-expression networks in adult brain were generated by removing lowly expressed genes (expression value < 0.3) and then retaining top 500,000 significant co-expression pairs between genes (FDR < 0.01, Pearson correlation test) using gene expression data from the adult human brain [55].

### Network analysis and visualization

To uncover disorder-specific topologies associated with BAG, subnetworks corresponding to each disorder and BAG-associated genes were extracted using the criterion that one side of the edge is a disorder risk gene or BAG-associated gene. The maximal connectivity subgraph was then extracted as the disorder-specific topology. The connectivity score (*C_s_*) was used to estimate the strength of the connections within the molecular network, defined as follows:


(5) $${\text{C}}_{s}=\frac{C_{i}/C_{o}}{N_{i}/N_{o}}$$

where $C_{i}$ represents the number of connections between a node with other nodes within a subset, $C_{o}$ represents the number of all the connections involving this node in the network, $N_{i}$ represents the number of nodes in the subset, and $N_{o}$ represents the number of all nodes in the molecular network. $C_{s}$ > 1 means that the node has stronger connections to the inside than to the outside.

To estimate the correlation of two subnetworks, such as a BAG-associated subnetwork and an ADHD-related subnetwork, the Jaccard distance index was employed to measure the enrichment between the networks, defined as follows:


(6) $$J(A,B)=\frac{A\cap B}{A\cup B}$$

where *A* is the node set of the BAG-associated subnetwork and *B* is the node set of the ADHD-related subnetwork.

For better visualization, we created nodes for each BAG-associated gene and other risk genes, connecting all nodes via transcriptional regulation or PPI. Finally, we arranged the networks using a perfuse circle layout. In the figure, we only presented the BAG-associated subnetworks. Network visualization was performed using Cytoscape [135].

## Code availability

The code for this study is available at GitHub (https://github.com/ZhaoXM-Lab/BrainAgepredict). The code has also been submitted to BioCode at the National Genomics Data Center (NGDC), China National Center for Bioinformation (CNCB) (BioCode: BT007788), which is publicly accessible at https://ngdc.cncb.ac.cn/biocode/tool/BT007788.

## Supplementary Material

qzaf064_Supplementary_Data

## Data Availability

The GWAS summary statistics dataset of BAG generated in this study has been deposited in the GWAS Catalog (GWAS Catalog: GCST90558034), which is publicly accessible at https://www.ebi.ac.uk/gwas/studies/GCST90558034. The GWAS summary statistics dataset of BAG has also been deposited in the Open Archive for Miscellaneous Data [136] at the National Genomics Data Center (NGDC), China National Center for Bioinformation (CNCB) (OMIX: OMIX015467), and is publicly accessible at https://ngdc.cncb.ac.cn/omix.

## CRediT author statement

**Xingzhong Zhao:** Methodology, Software, Formal analysis, Visualization, Writing – original draft, Writing – review & editing. **Anyi Yang:** Formal analysis. **Jing Ding:** Supervision, Writing – review & editing. **Yucheng T. Yang:** Conceptualization, Supervision, Writing – original draft, Writing – review & editing. **Xing-Ming Zhao:** Conceptualization, Supervision, Writing – original draft, Writing – review & editing. All authors have read and approved the final manuscript.
