# Supplementary material for: Regulatory Genomic Circuitry of Brain Age by Integrative Functional Genomic Analyses
Source: Genomics Proteomics Bioinformatics. 2025 Aug 8;23(5):qzaf064. doi: 10.1093/gpbjnl/qzaf064 (PMC12996886; doi:10.1093/gpbjnl/qzaf064)
Supplement: qzaf064_Supplementary_Data [file qzaf064_supplementary_data.zip › supplementary material captions.docx]

**Supplementary figures**

**Figure S1 Schematic showing the framework of ACN model**

The channel numbers of 3D CNN blocks in the encoder are shown in red. The ACN baseline model is depicted in the red boxes. 3D CNN, three-dimensional convolutional neural network.

**Figure S2 *t*-SNE plot showing the stratified features after encoder in the training set**

Each point represents an individual in the training set (*N* = 1407). Clustering patterns of individuals based on the ACN baseline models (left panel) and ACN models (right panel) are visualized. In each panel, we compare the clustering performance of adding a mixed module of data site (upper) and gender (lower) to the baseline model or not.

**Figure S3 *t*-SNE plot showing the stratified features after encoder in the testing set**

Each point represents an individual in the testing set (*N* = 403). Clustering patterns of individuals based on the ACN baseline models (left panel) and ACN models (right panel) are visualized. In each panel, we compare the clustering performance of adding a mixed module of data site (upper) and gender (lower) to the baseline model or not.

**Figure S4 Prediction accuracy of AD participants based on the stratified features**

The classification of AD participants from the ADNI dataset is performed using the SVM algorithm with 5-fold cross-validation procedure. The blue line represents the mean classification accuracy, and the red dashed line represents the random chance. The shaded area represents ±1 standard deviation of the true positive rate (TPR) at each false positive rate (FPR) among the 5-fold cross-validation. SVM, support vector machine; ROC, receiver operating characteristic; AUC, area under the ROC curve; std. dev., standard deviation.

**Figure S5 Correlation between the predicted ages and chronological ages**

Each point represents an individual from the UKB cohort. The correlation is evaluated using the Pearson correlation coefficient. UKB, UK Biobank; MAE, mean absolute error.

**Figure S6 Regional plots for each of the ten lead SNPs**

The genes located within each local region are shown. Colors indicate varying linkage disequilibrium r^2^ values. SNP, single nucleotide polymorphism.

**Figure S7 Partitioned heritability enrichment of BAG GWAS in functional genomic regions**

The red dashed line indicates an FDR threshold at 5% level.

**Figure S8 Odds ratio plot showing MR results from brain disorders to BAG**

The row represents the different methods of MR analysis. The odds ratio larger than one means that disorders can accelerate brain aging.

**Figure S9 Leave-one-out plot in the MR results from disorders to BAG**

**A.** Leave-one-out plot of sensitivity analysis of single SNP effect on AD to BAG. **B.** Leave-one-out plot of sensitivity analysis of single SNP effect on BIP to BAG. The black point denotes the causal effect estimate of brain disorders on BAG after discarding a certain SNP, and the black line signifies the 95% CI of the estimate. The red point symbolizes the causal effect estimate of disorders on BAG the valid SNPs, and the red line indicates the 95% CI of the estimate. CI, confidence interval.

**Figure S10 Overlap between the prioritized BAG-associated genes based on different methods**

The BAG-associated genes are prioritized using MAGMA, eQTL, and chromatin interaction (Hi-C) data. MAGMA, Multi-marker Analysis of GenoMic Annotation; Hi-C, high-throughput chromosome conformation capture.

**Figure S11 BAG and MAG in individuals diagnosed with** **CN, EMCI, LMC, MCI, and AD**

**A.** and **B**. The BAG and MAG scores are predicted using the MRI images (A) and DNA methylation data (B), respectively. The statistical significance of the difference between MCI and AD groups is estimated using the Wilcoxon test. CN, cognitively normal; MCI, mild cognitive impairment; EMCI, early MCI; LMCI, late MCI; MRI, magnetic resonance imaging.

**Figure S12 Interaction networks of BAG-associated and MAG-associated genes among TRNs**

The BAG-associated and MAG-associated genes are shown in orange and grey, respectively.

**Figure S13 Interactions networks of BAG-associated genes and risk genes of brain disorders among TRNs**

The BAG-associated genes (purple), risk genes of brain disorders (green), and the shared ones (yellow) are shown in different colors.

**Figure S14 Interaction networks of BAG-associated genes and risk genes of brain disorders among gene co-expression networks**

The BAG-associated genes (purple), risk genes of brain disorders (green), and the shared ones (yellow) are shown in different colors.

**Figure S15 Interaction networks of BAG-associated genes and risk genes of brain disorders among PPI networks**

The BAG-associated genes (purple), risk genes of brain disorders (green), and the shared ones (yellow) are shown in different colors.

**Figure S16 Overlap between risk genes of brain disorders and the interacted genes with BAG-associated genes**

**A.** and **B.** Upset plot showing the overlap between risk genes of diverse brain disorders and the interacted genes among gene co-expression networks (A) and PPI networks (B).

**Figure S17 Pleiotropy between BAG-related genes and risk genes of brain disorders among gene co-expression network and PPI network active in human brain**

**A.** and **B.** Network showing the pleiotropy between BAG-associated gene and risk gene of diverse brain disorders among gene co-expression network (A) and PPI network (B). Diamonds represent BAG-associated genes, and circles represent risk genes of brain disorders.

**Figure S18 Correlation between the first principal component of the stratified features and chronological ages**

**A.** and **B.** Each point represents an individual in the testing set (*N* = 403). The stratified features from the ACN baseline model (A) and the ACN model (B) are used for computing their first principal component.

**Table S1**  **The demographic information for model training and testing in this study**

**Table S2 Ablation experiments with different loss functions for the baseline model**

**Table S3 The genetic variants that exceeded genome-wide significance (*P* < 5E−08) of BAG**

**Table S4 The lead SNPs of BAG after LD clumping**

**Table S5 The replication of lead SNPs in previous studies**

**Table S6 The detailed names of cell types**

**Table S7 Source and information of the GWAS summary statistics used in this study**

**Table S8 Pleiotropy test for MR results**

**Table S9 Heterogeneity test for MR results**

**Table S10 MR results using BAG as the outcome and the 13 complex traits as the exposure**

**Table S11 MR results using the 13 complex traits as the outcome and BAG as the exposure**

**Table S12 BAG-associated genes prioritized based on different methods**

**Table S13 Collection of known risk genes of seven brain disorders**

**Table S14 Reconstructed PPI networks active in human brain**
