# Supplementary figures and images for: Regulatory Genomic Circuitry of Brain Age by Integrative Functional Genomic Analyses
Source: Genomics Proteomics Bioinformatics. 2025 Aug 8;23(5):qzaf064. doi: 10.1093/gpbjnl/qzaf064 (PMC12996886; doi:10.1093/gpbjnl/qzaf064)

**A**

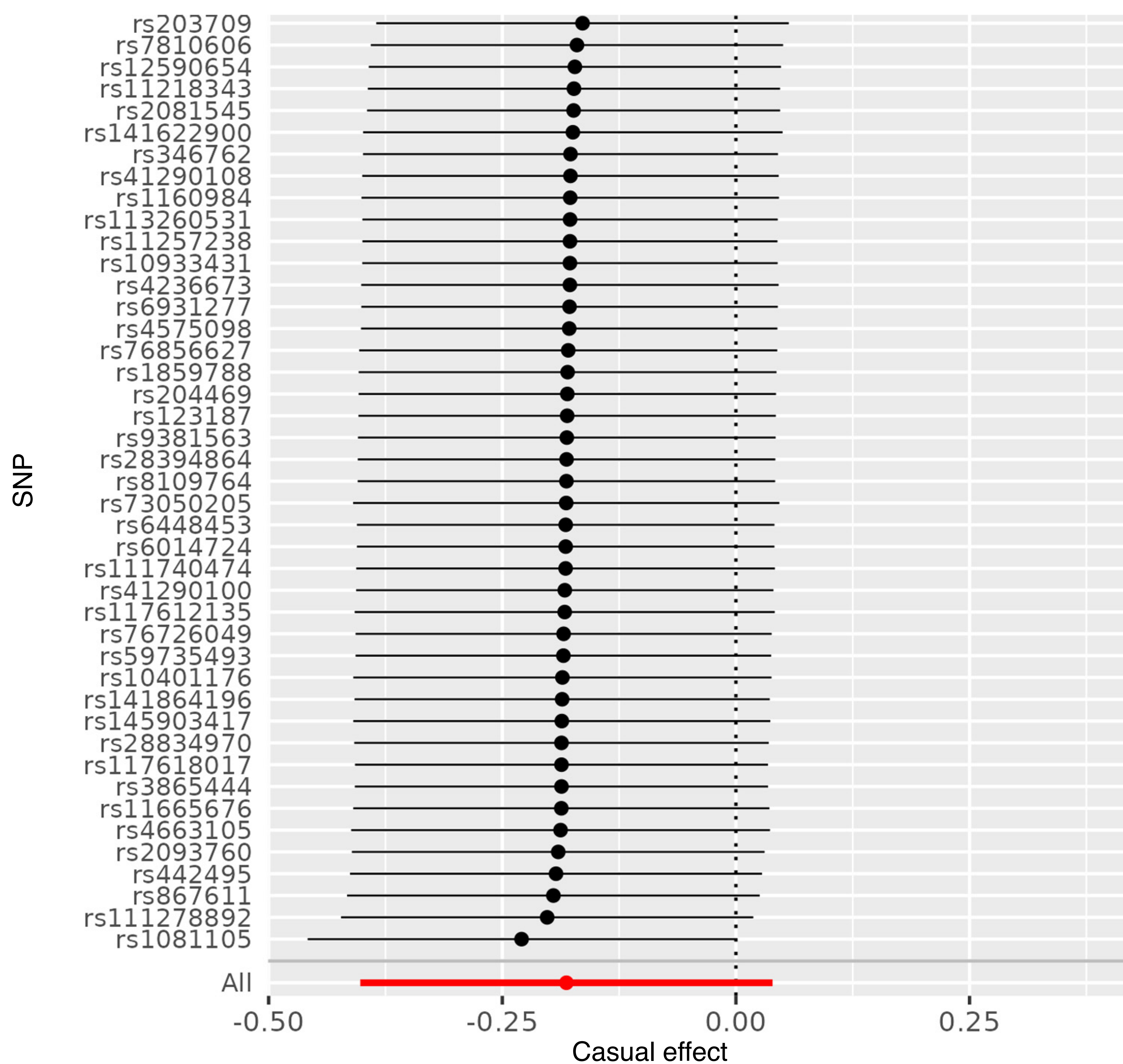

# B

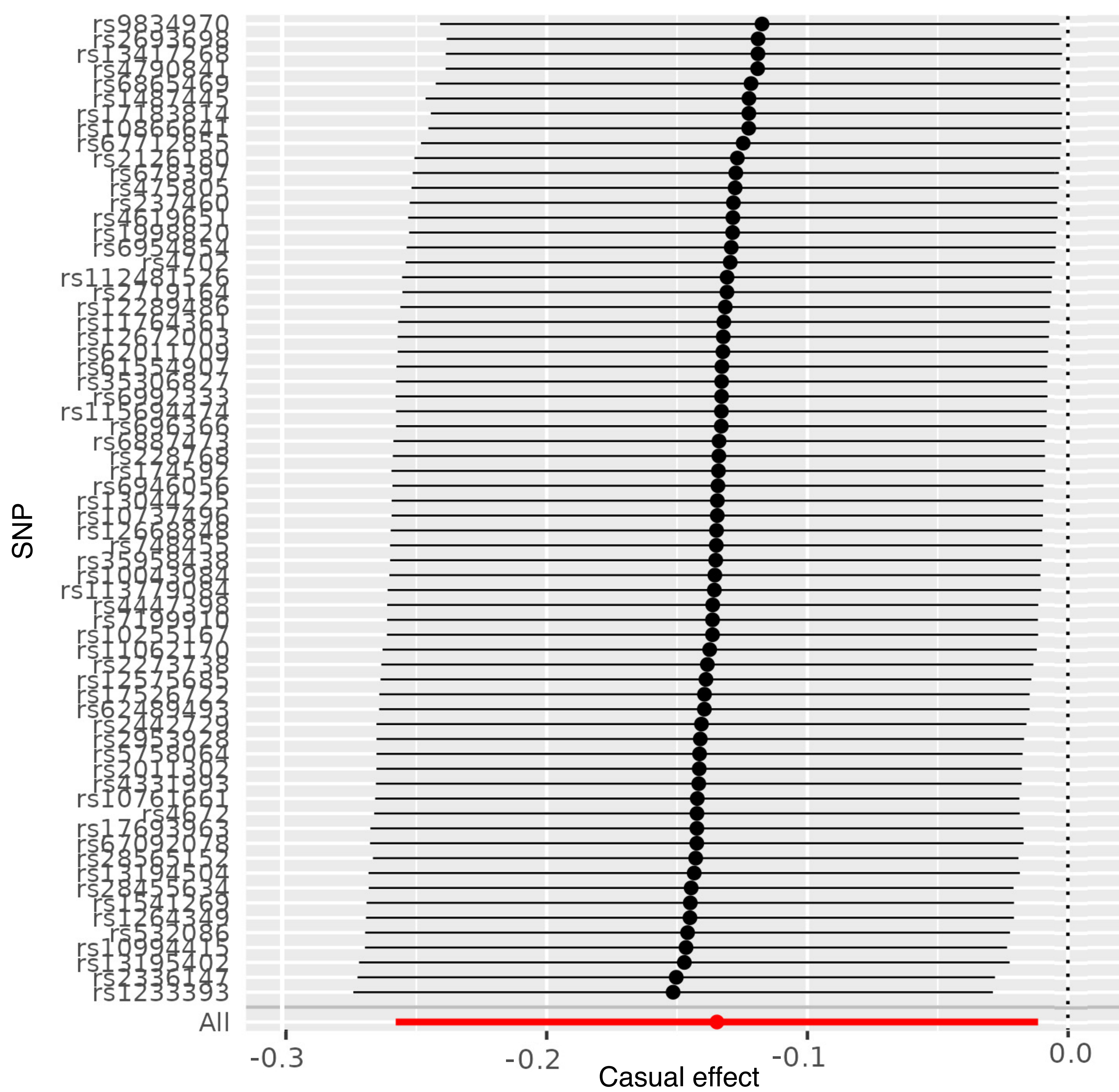

Supplement: qzaf064_Supplementary_Data [file qzaf064_supplementary_data.zip › FIgure S9.pdf]

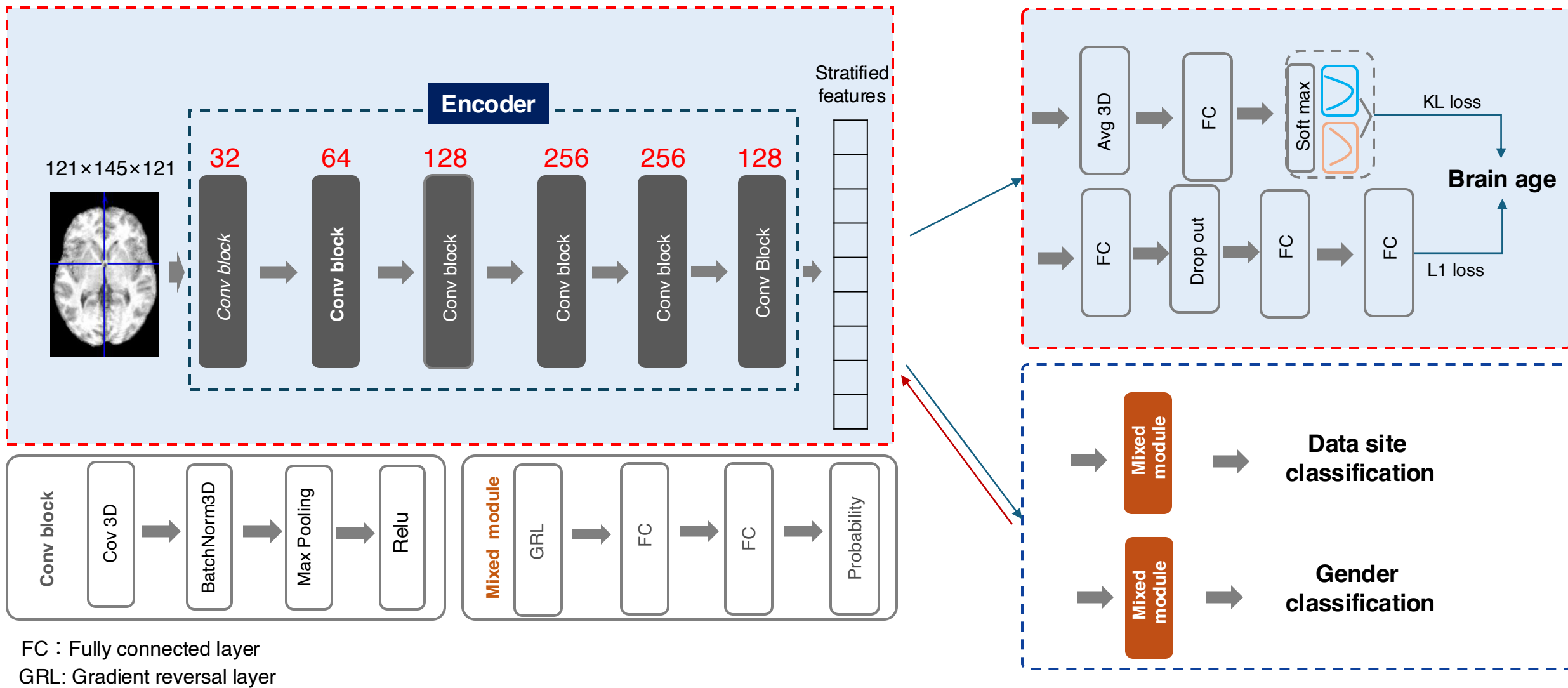

Supplement: qzaf064_Supplementary_Data [file qzaf064_supplementary_data.zip › Figure S1.pdf]

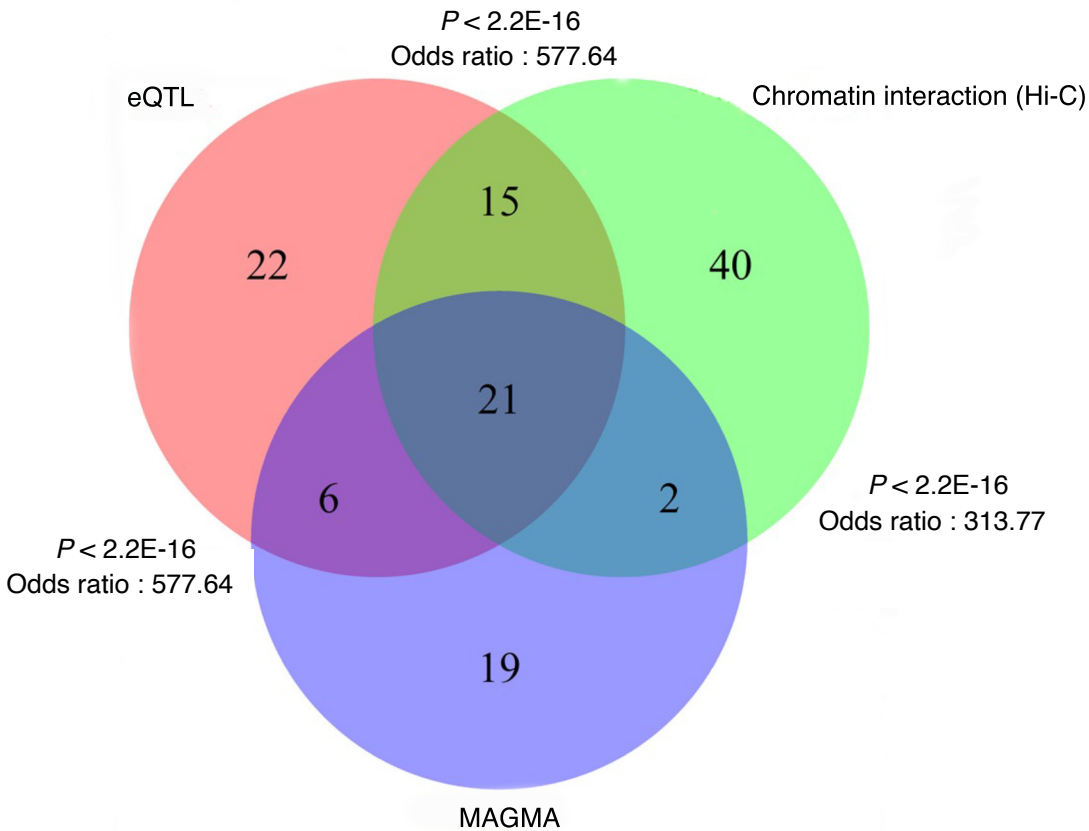

Supplement: qzaf064_Supplementary_Data [file qzaf064_supplementary_data.zip › Figure S10.pdf]

A

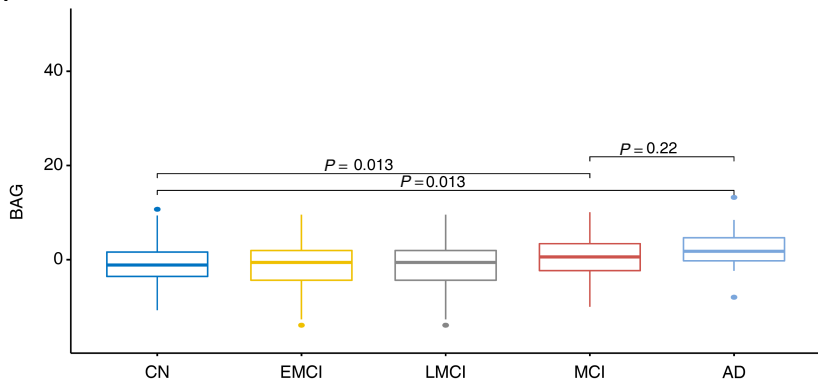

B

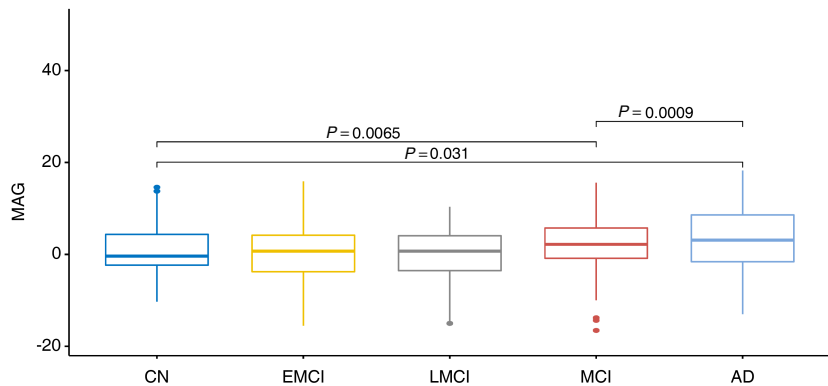

Supplement: qzaf064_Supplementary_Data [file qzaf064_supplementary_data.zip › Figure S11.pdf]

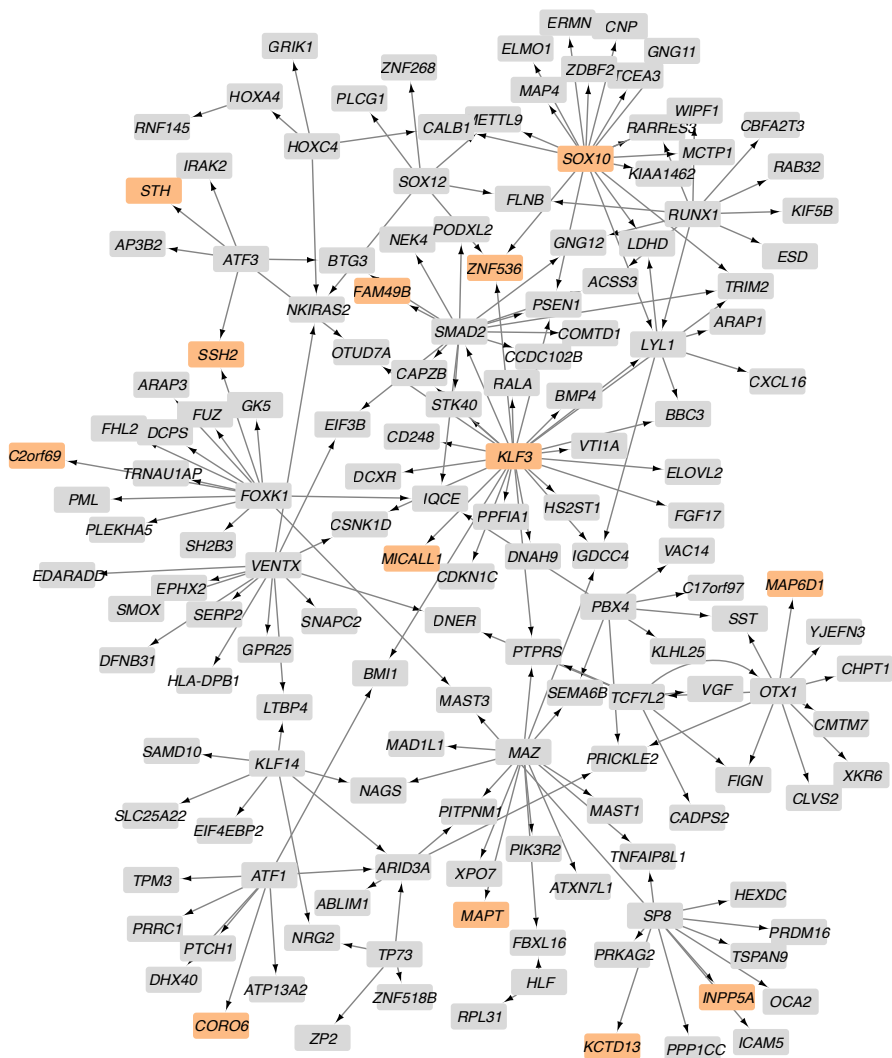

Supplement: qzaf064_Supplementary_Data [file qzaf064_supplementary_data.zip › Figure S12.pdf]

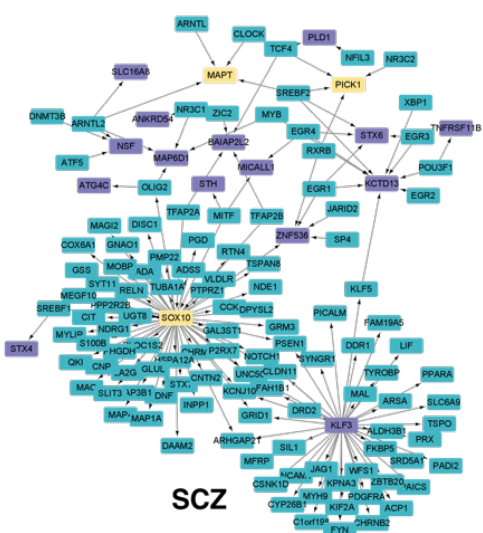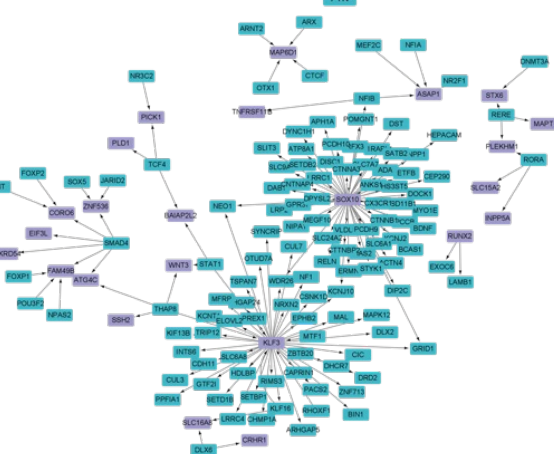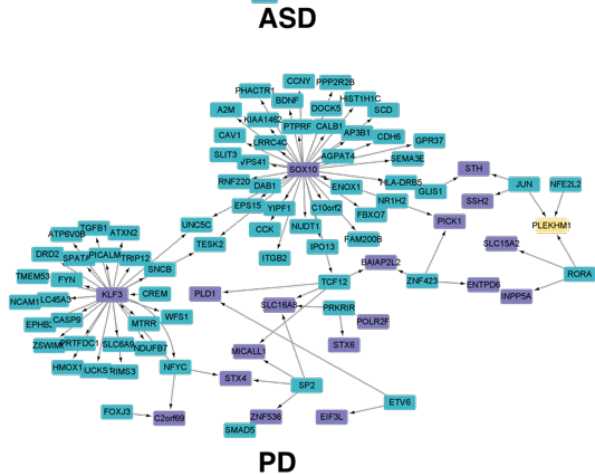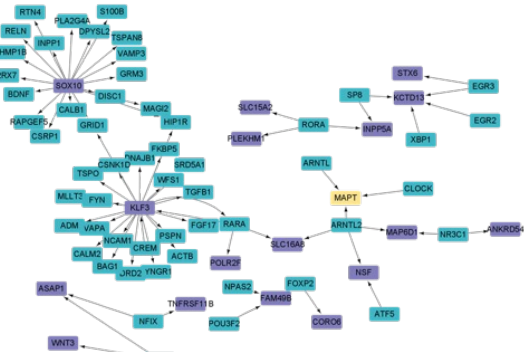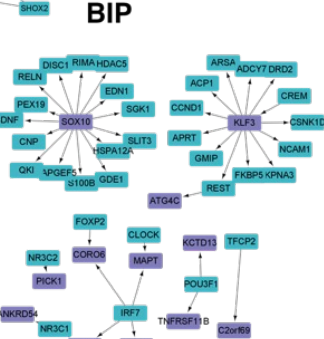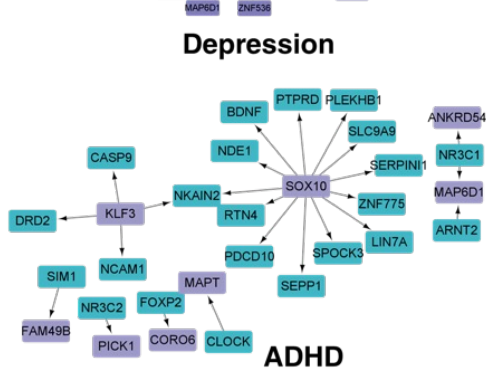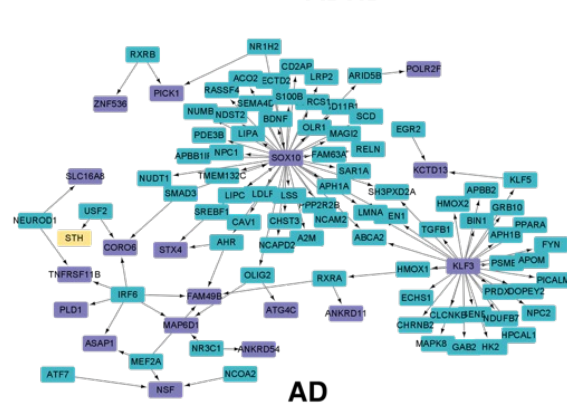

Supplement: qzaf064_Supplementary_Data [file qzaf064_supplementary_data.zip › Figure S13.pdf]

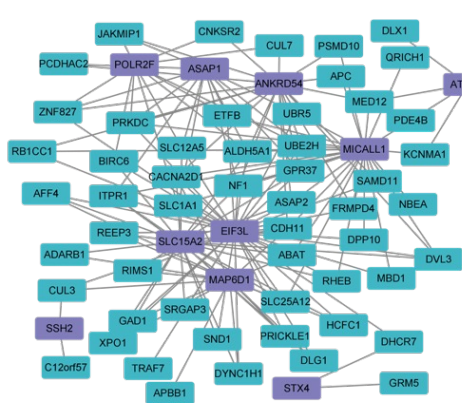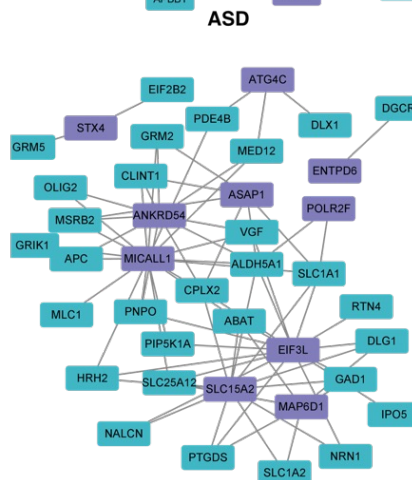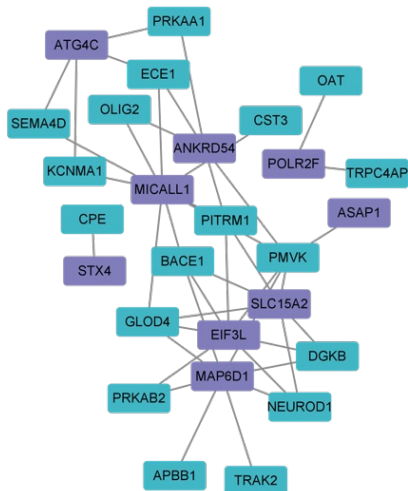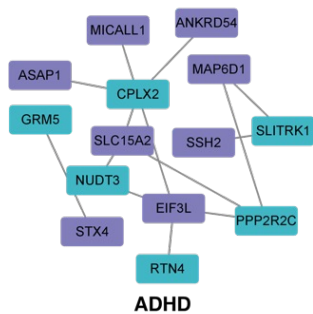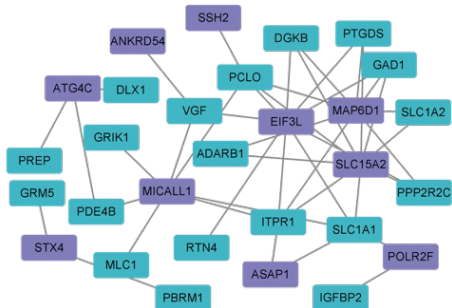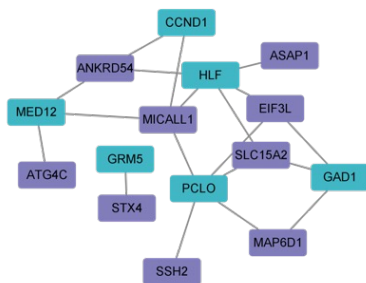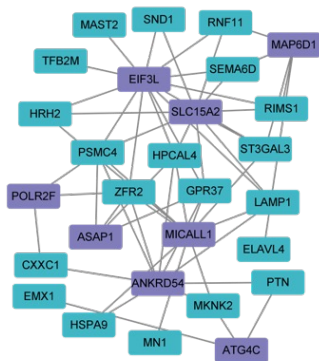

Supplement: qzaf064_Supplementary_Data [file qzaf064_supplementary_data.zip › Figure S14.pdf]

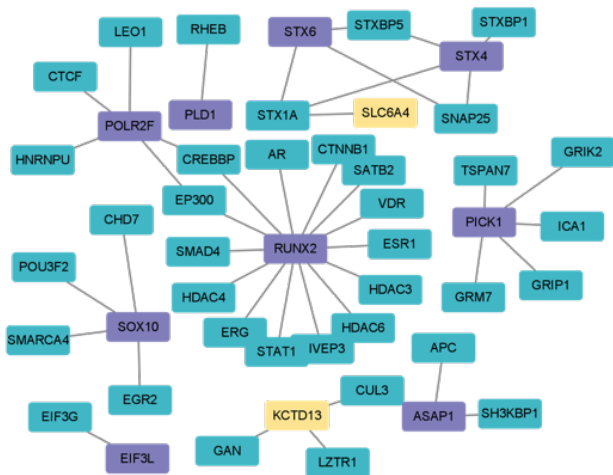

**ASD**

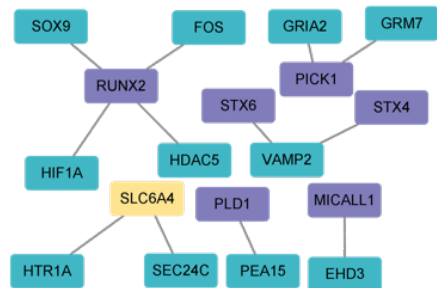

**MDD**

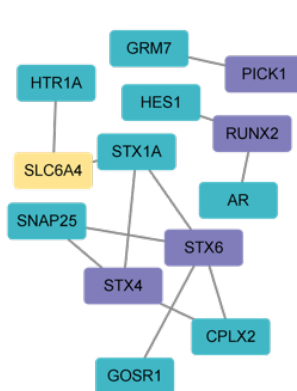

**ADHD**

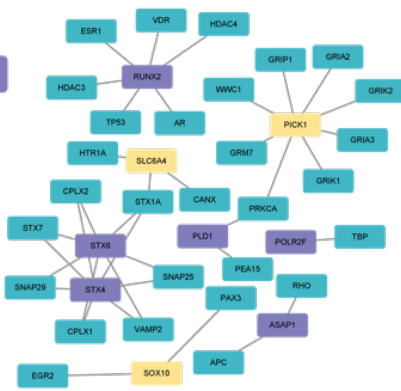

**SCZ**

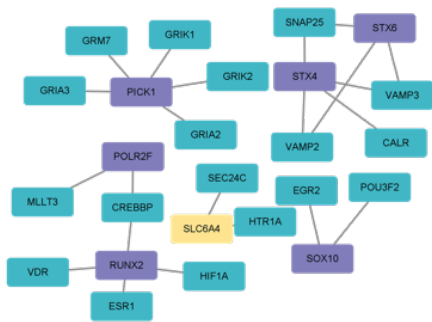

**BIP**

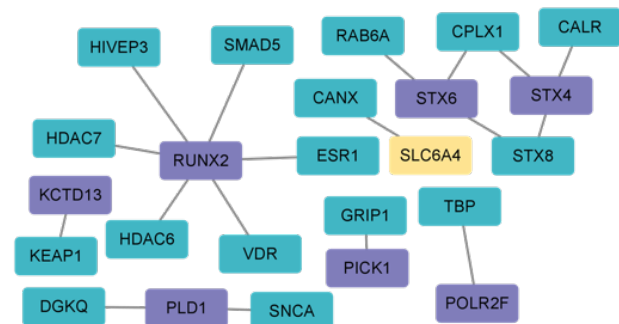

**PD**

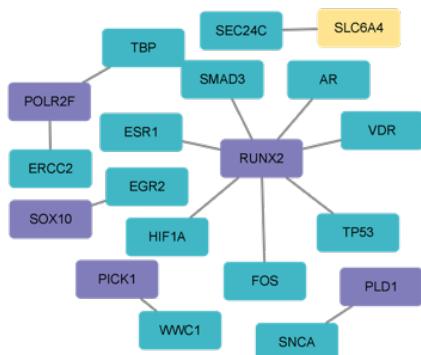

**AD**

Supplement: qzaf064_Supplementary_Data [file qzaf064_supplementary_data.zip › Figure S15.pdf]

**A**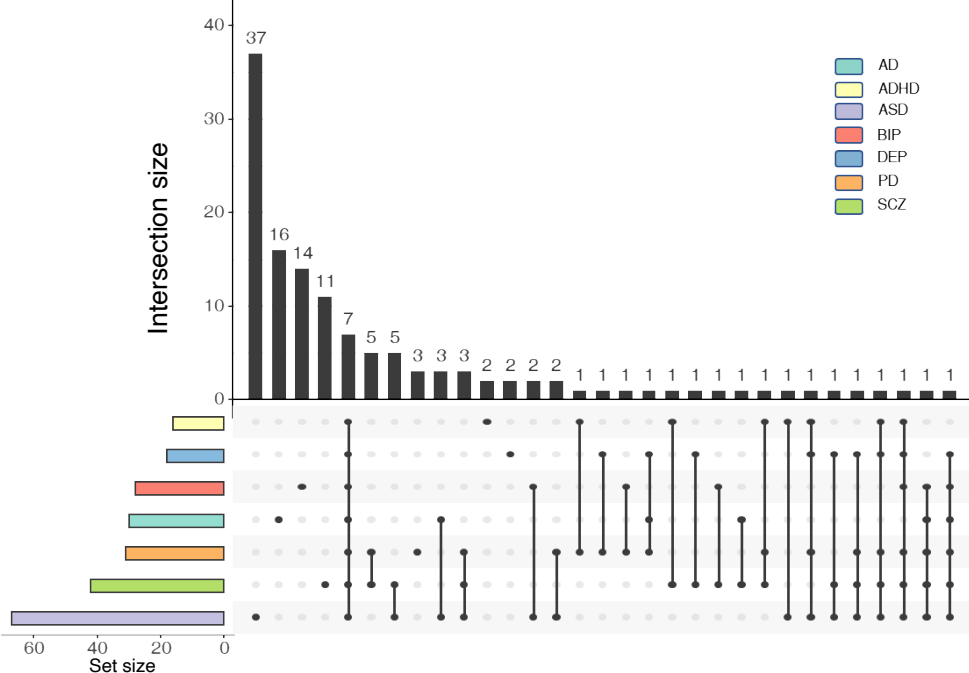**B**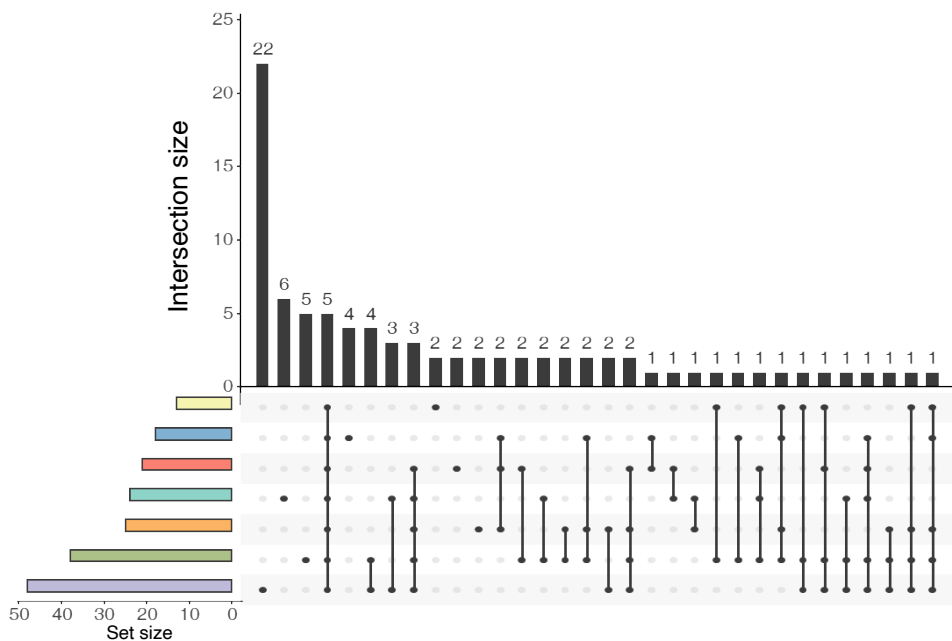

Supplement: qzaf064_Supplementary_Data [file qzaf064_supplementary_data.zip › Figure S16.pdf]

A

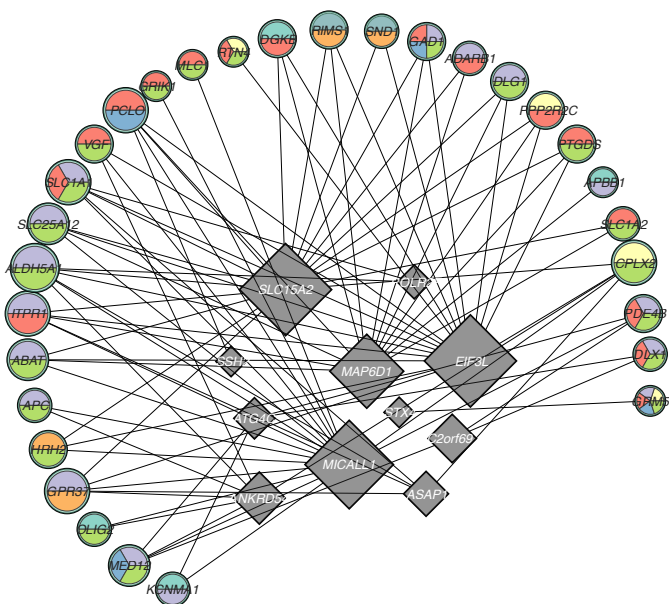

B

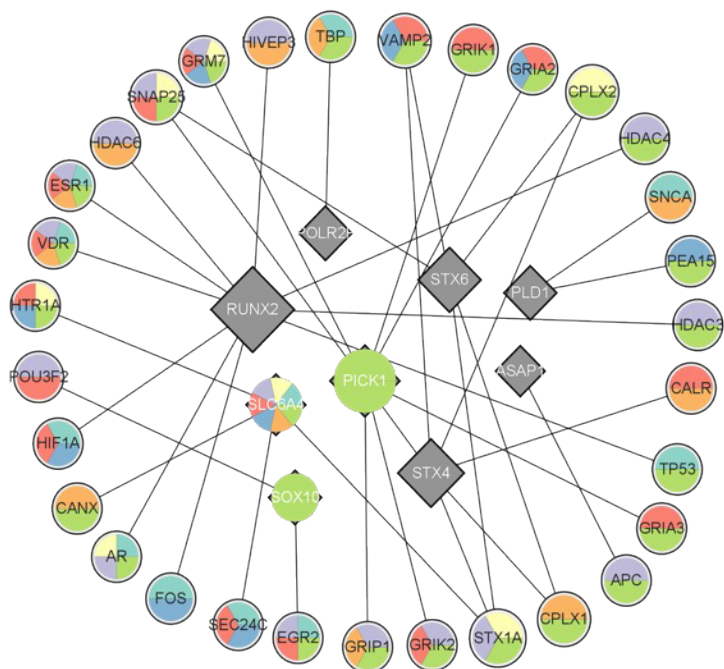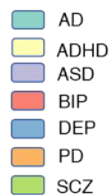

Supplement: qzaf064_Supplementary_Data [file qzaf064_supplementary_data.zip › Figure S17.pdf]

**A**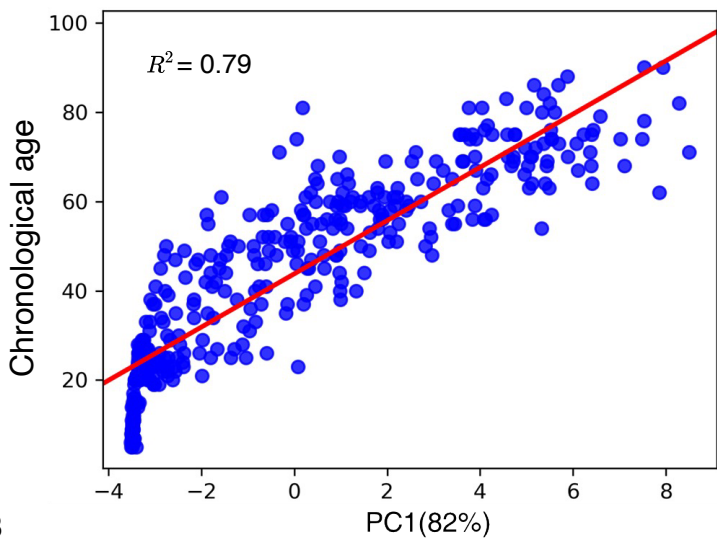**B**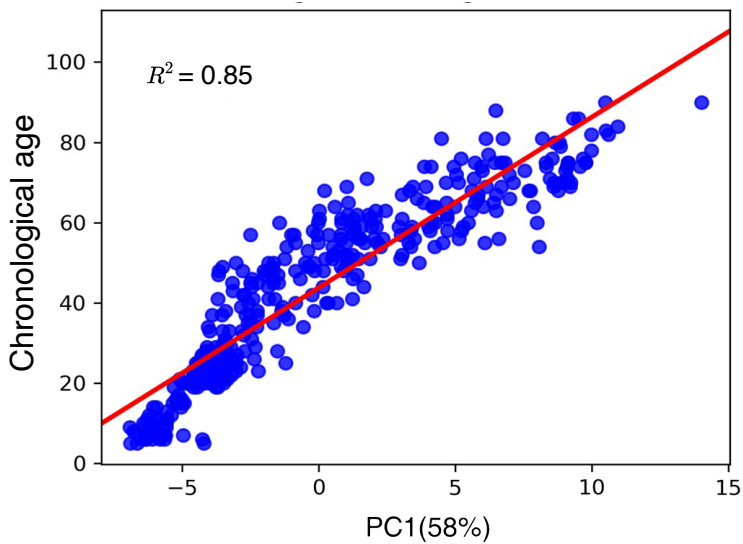

Supplement: qzaf064_Supplementary_Data [file qzaf064_supplementary_data.zip › Figure S18.pdf]

**ACN baseline model**

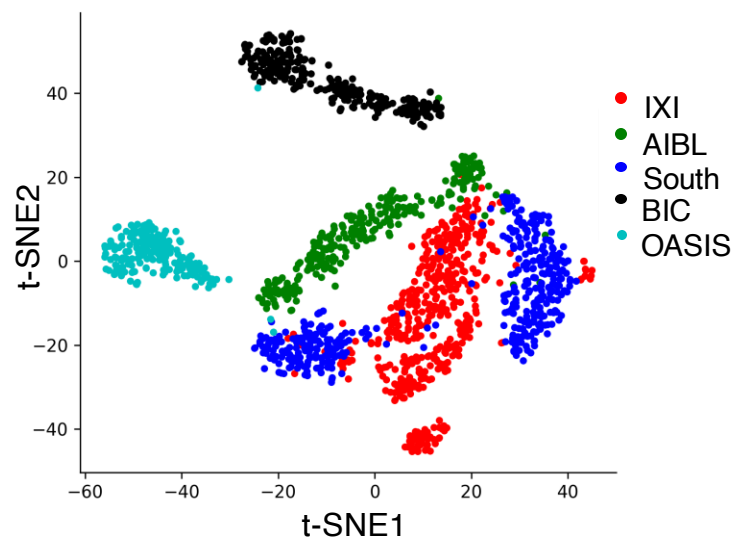

**ACN model**

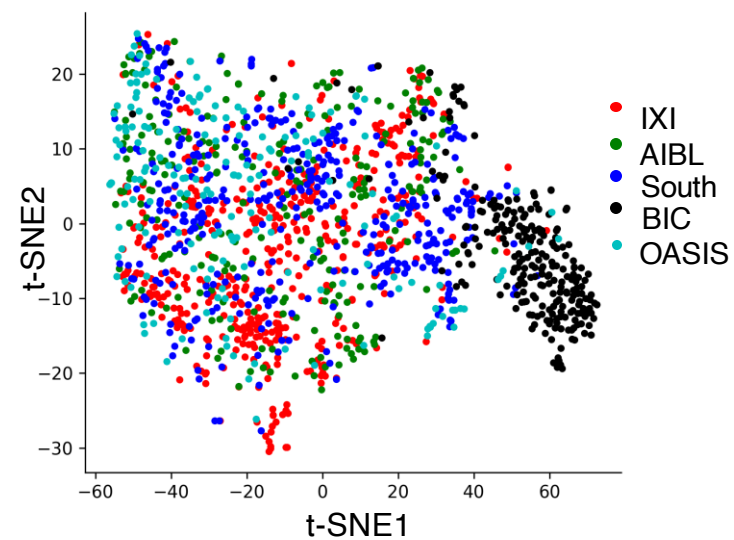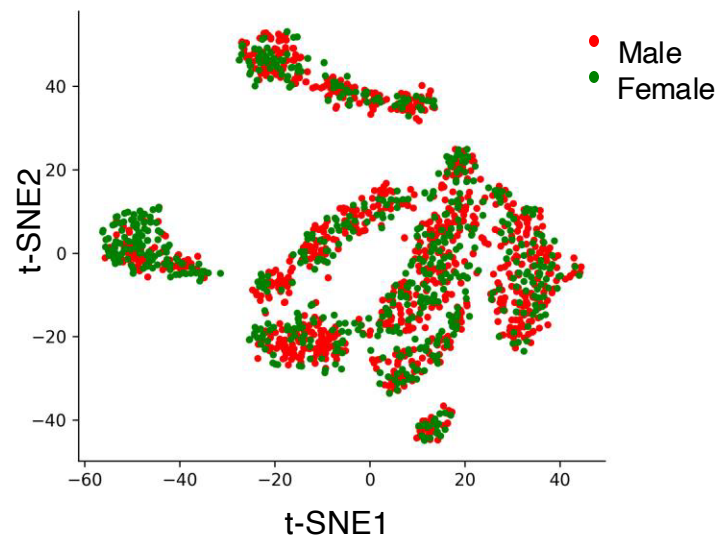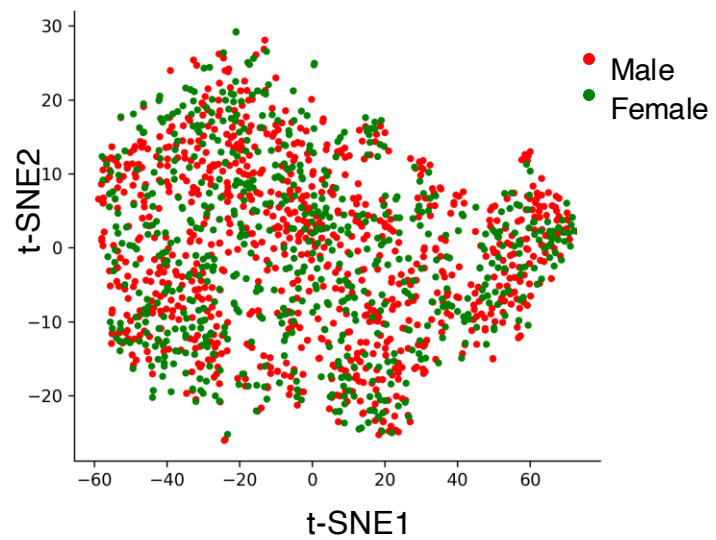

Supplement: qzaf064_Supplementary_Data [file qzaf064_supplementary_data.zip › Figure S2.pdf]

### ACN baseline model

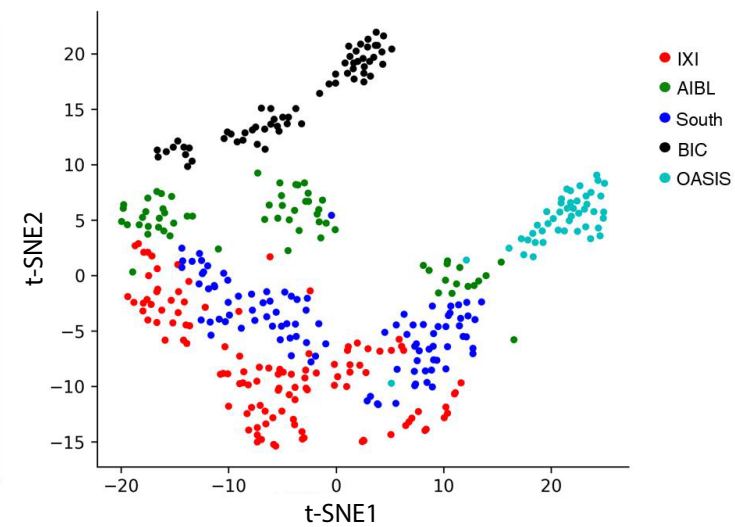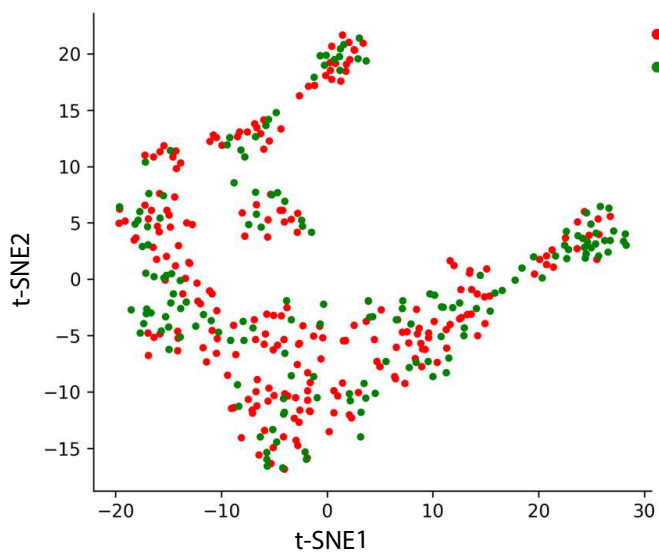

### ACN model

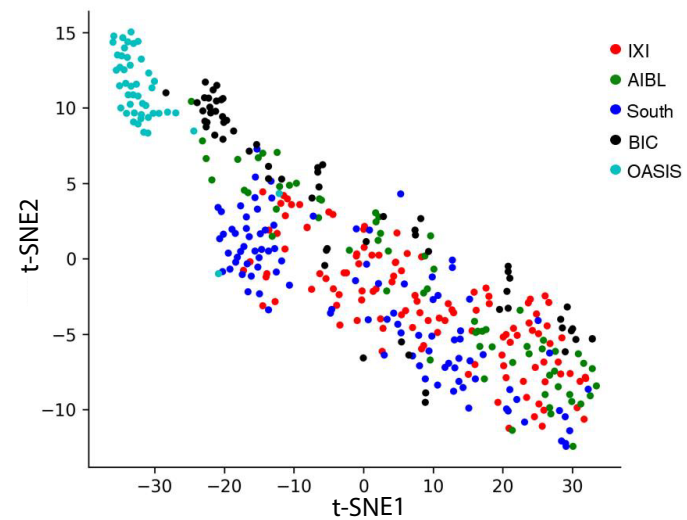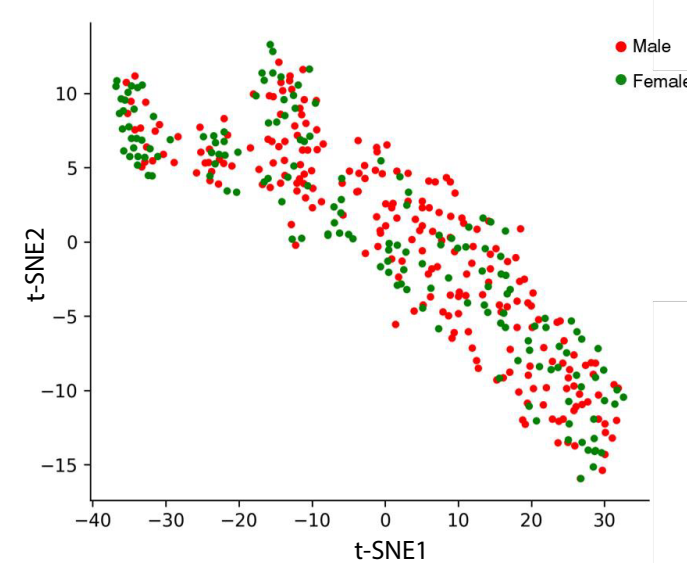

Supplement: qzaf064_Supplementary_Data [file qzaf064_supplementary_data.zip › Figure S3.pdf]

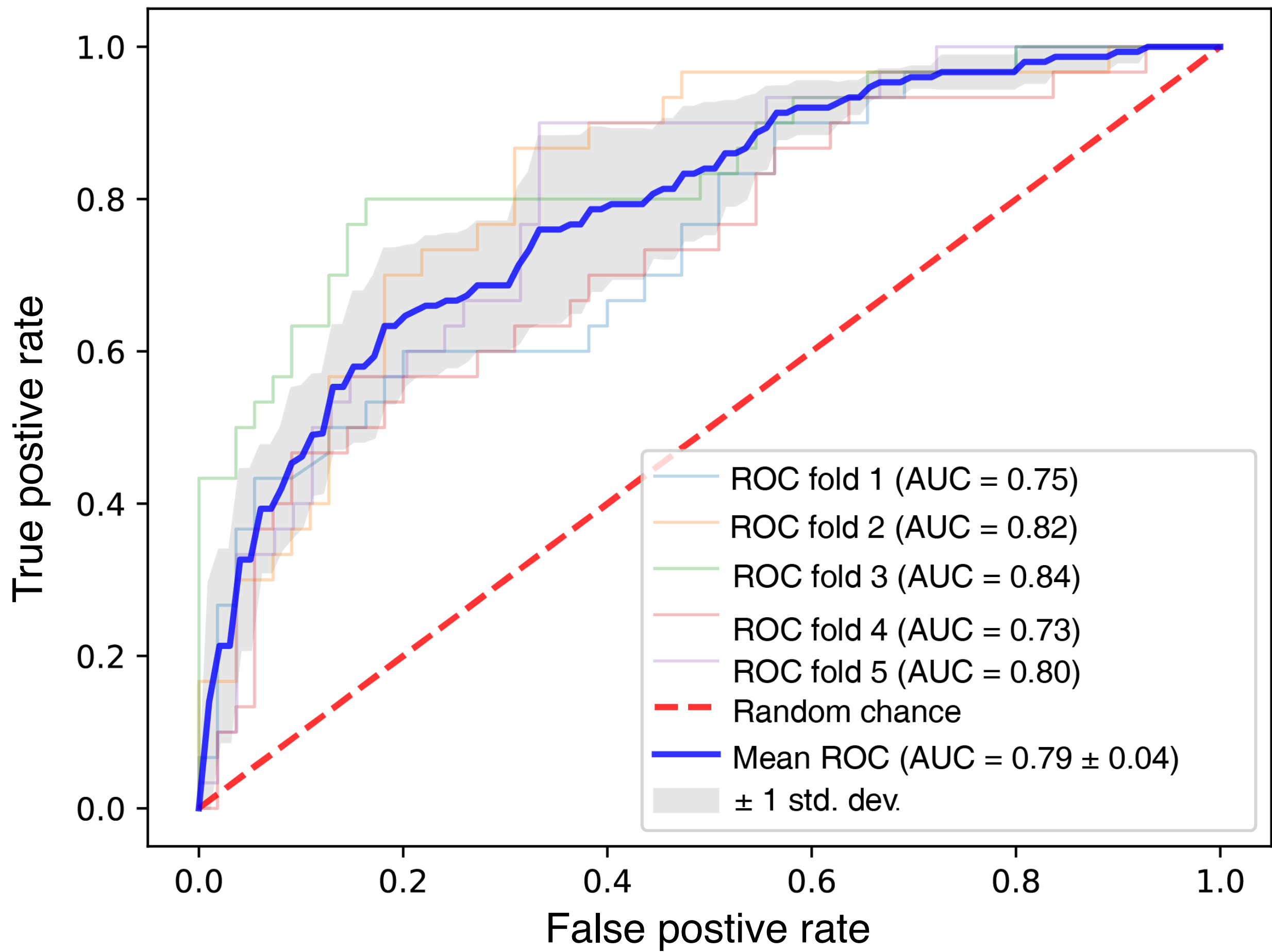

Supplement: qzaf064_Supplementary_Data [file qzaf064_supplementary_data.zip › Figure S4.pdf]

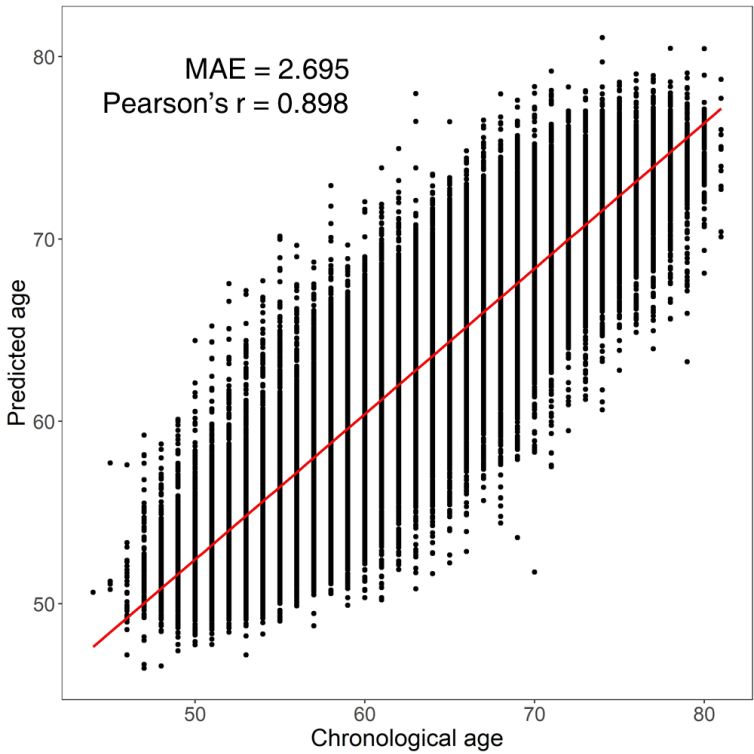

Supplement: qzaf064_Supplementary_Data [file qzaf064_supplementary_data.zip › Figure S5.pdf]

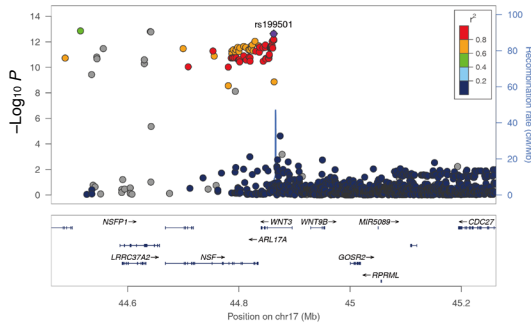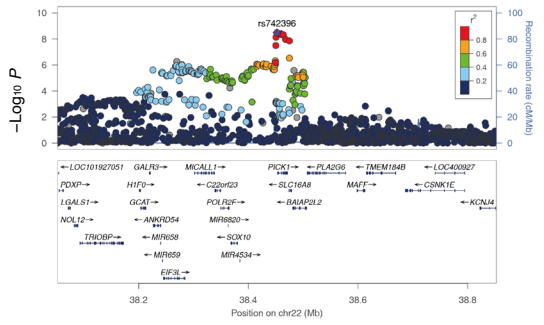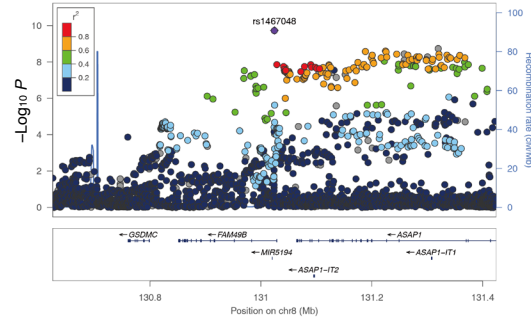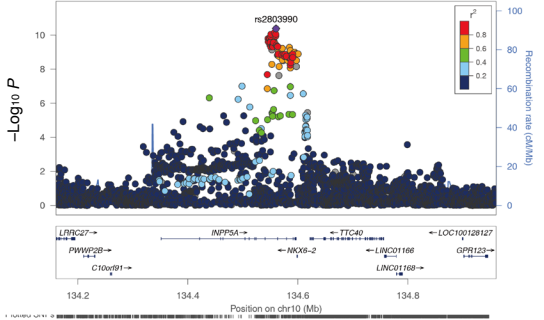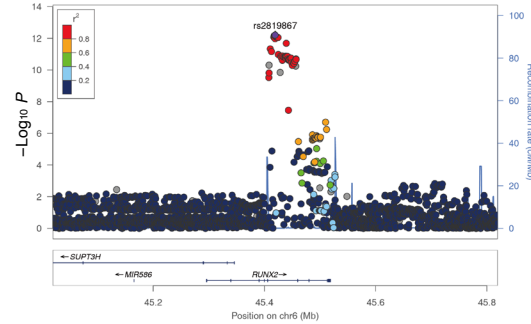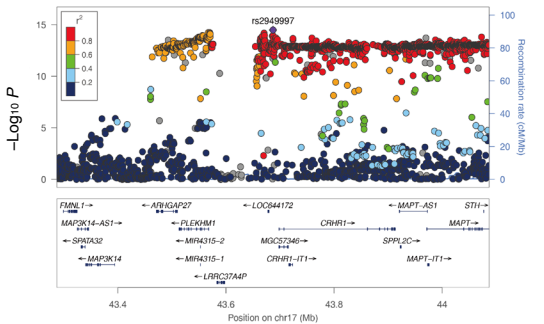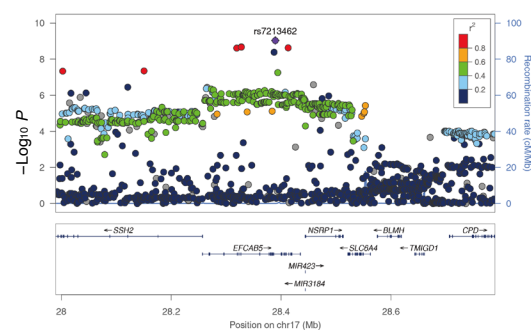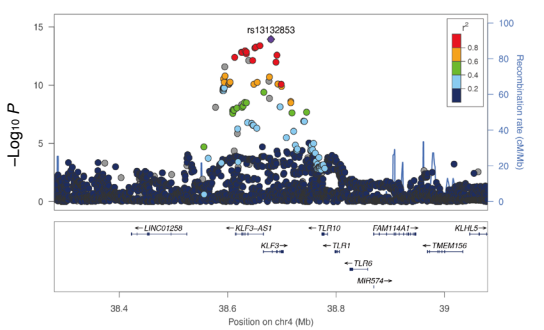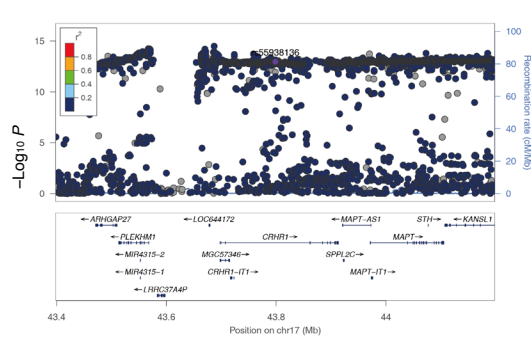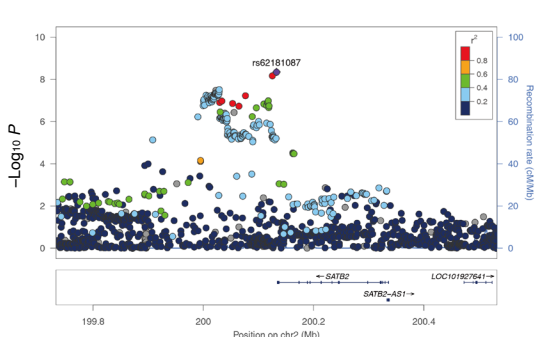

Supplement: qzaf064_Supplementary_Data [file qzaf064_supplementary_data.zip › Figure S6.pdf]

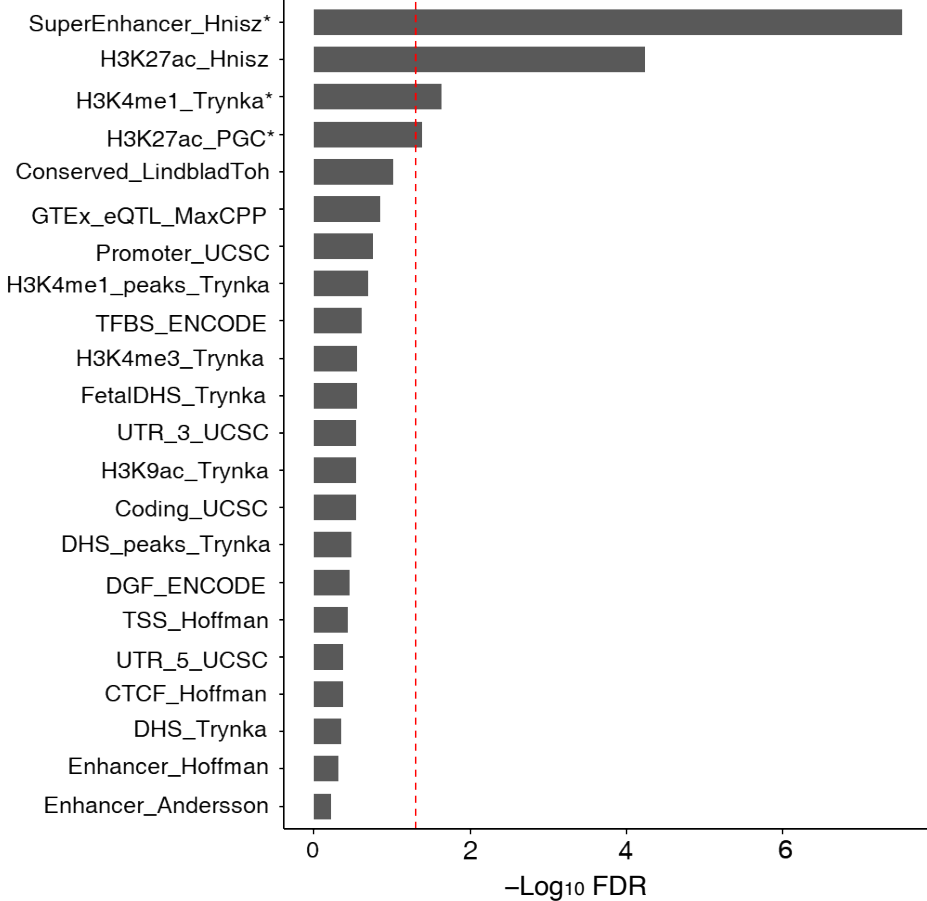

Supplement: qzaf064_Supplementary_Data [file qzaf064_supplementary_data.zip › Figure S7.pdf]

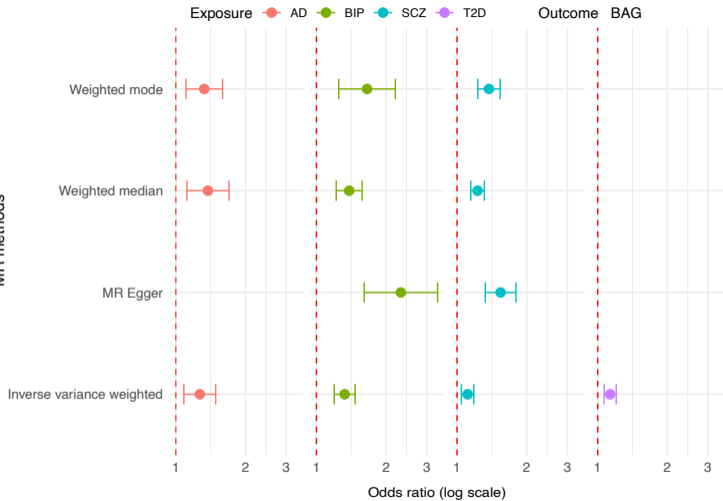

Supplement: qzaf064_Supplementary_Data [file qzaf064_supplementary_data.zip › Figure S8.pdf]
